# Safety and Immunogenicity of Therapeutic DNA Vaccination in Individuals Treated with Antiretroviral Therapy during Acute/Early HIV-1 Infection

**DOI:** 10.1371/journal.pone.0010555

**Published:** 2010-05-10

**Authors:** Eric S. Rosenberg, Barney S. Graham, Ellen S. Chan, Ronald J. Bosch, Vicki Stocker, Janine Maenza, Martin Markowitz, Susan Little, Paul E. Sax, Ann C. Collier, Gary Nabel, Suzanne Saindon, Theresa Flynn, Daniel Kuritzkes, Dan H. Barouch, for the ACTG A5187 Team

**Affiliations:** 1 Massachusetts General Hospital and Harvard Medical School, Boston, Massachusetts, United States of America; 2 Vaccine Research Center, National Institute of Allergy and Infectious Diseases, National Institutes of Health, Bethesda, Maryland, United States of America; 3 Harvard School of Public Health, Boston, Massachusetts, United States of America; 4 Social and Scientific Systems, Silver Spring, Maryland, United States of America; 5 University of Washington, Seattle, Washington, United States of America; 6 Aaron Diamond AIDS Research Center, Rockefeller University, New York, New York, United States of America; 7 University of California San Diego, San Diego, California, United States of America; 8 Brigham and Women's Hospital and Harvard Medical School, Boston, Massachusetts, United States of America; 9 Beth Israel Deaconess Medical Center, Harvard Medical School, Boston, Massachusetts, United States of America; University of Toronto, Canada

## Abstract

**Background:**

An effective therapeutic vaccine that could augment immune control of HIV-1 replication may abrogate or delay the need for antiretroviral therapy. AIDS Clinical Trials Group (ACTG) A5187 was a phase I/II, randomized, placebo-controlled, double-blinded trial to evaluate the safety and immunogenicity of an HIV-1 DNA vaccine (VRC-HVDNA 009-00-VP) in subjects treated with antiretroviral therapy during acute/early HIV-1 infection. (clinicaltrials.gov NCT00125099)

**Methods:**

Twenty healthy HIV-1 infected subjects who were treated with antiretroviral therapy during acute/early HIV-1 infection and had HIV-1 RNA<50 copies/mL were randomized to receive either vaccine or placebo. The objectives of this study were to evaluate the safety and immunogenicity of the vaccine. Following vaccination, subjects interrupted antiretroviral treatment, and set-point HIV-1 viral loads and CD4 T cell counts were determined 17–23 weeks after treatment discontinuation.

**Results:**

Twenty subjects received all scheduled vaccinations and discontinued antiretroviral therapy at week 30. No subject met a primary safety endpoint. No evidence of differences in immunogenicity were detected in subjects receiving vaccine versus placebo. There were also no significant differences in set-point HIV-1 viral loads or CD4 T cell counts following treatment discontinuation. Median set-point HIV-1 viral loads after treatment discontinuation in vaccine and placebo recipients were 3.5 and 3.7 log_10_ HIV-1 RNA copies/mL, respectively.

**Conclusions:**

The HIV-1 DNA vaccine (VRC-HIVDNA 009-00-VP) was safe but poorly immunogenic in subjects treated with antiretroviral therapy during acute/early HIV-1 infection. Viral set-points were similar between vaccine and placebo recipients following treatment interruption. However, median viral load set-points in both groups were lower than in historical controls, suggesting a possible role for antiretroviral therapy in persons with acute or early HIV-1 infection and supporting the safety of discontinuing treatment in this group.

**Trial Registration:**

Clinicaltrials.gov NCT00125099

## Introduction

Despite the striking decline in morbidity and mortality in persons receiving antiretroviral therapy [1], the short- and long-term toxicities, increasing drug resistance, challenges with adherence, and cost make the prospect of long-term therapy difficult for many HIV-1 infected individuals. More importantly, the majority of HIV-1 infected individuals live in developing countries with limited access to antiretroviral therapy. An effective therapeutic vaccine that could induce or augment HIV-1-specific immune responses may potentially delay or reduce the need for antiretroviral therapy.

One approach to inducing HIV-1-specific immunity is through the delivery of multiple viral antigens by DNA plasmids. The DNA vaccine VRC-HIVDNA009-00-VP is a 4 plasmid mixture encoding a subtype B Gag-Pol-Nef fusion protein and modified envelope (Env) constructs from HIV-1 subtypes A, B and C. This multiclade DNA vaccine has previously been evaluated in a phase I dose escalation study in healthy, HIV-1-uninfected adults and was found to be safe and well tolerated[2]. Furthermore, the vaccine induced significant cellular and humoral immune responses. Because this vaccine appeared safe and immunogenic in HIV-1-uninfected adults, we assessed the potential utility of this vaccine in healthy HIV-1-infected individuals.

Here we report the findings from ACTG A5187, a phase I/II, randomized, placebo-controlled, double-blinded trial of the DNA vaccine VRC-HIVDNA009-00-VP (clinicaltrials.gov: NCT00125099). The first phase of this study was to evaluate the safety of the vaccine, which was the primary aim. The second phase of the study was to determine if there was a difference between the two treatment arms in HIV-1 RNA levels and CD4 T cell counts at viral load set-point after antiretroviral therapy was discontinued. Exploratory analyses assessed the immunogenicity of the vaccine. The study enrolled 20 healthy, HIV-1 infected subjects who were treated with antiretroviral therapy during acute or early infection. The rationale for studying persons treated during acute or early HIV-1 infection was to test this vaccine in persons presumed to have relatively preserved immune function[3], [4]. Furthermore, it was felt that antiviral treatment interruption would likely be safe and well tolerated in this group [5]. A concurrently randomized placebo arm was used to estimate vaccine efficacy [6].

## Materials and Methods

The protocol for this trial and supporting CONSORT checklist are available as supporting information; see Checklist S1 and Protocol S1.

### Participant Selection

Twenty healthy HIV-1 infected adults, aged 26–47, who were treated with antiretroviral therapy during acute or early HIV-1 infection participated in this study (one additional subject was randomized but withdrew from the study before the first injection and was therefore replaced, per protocol). Subjects with treated acute HIV-1 infection were defined as initiating antiretroviral therapy after being diagnosed by a positive HIV-1 viral load and either a negative or indeterminate Western blot. Early infection was defined as having a positive ELISA or a positive Western blot with a non-reactive detuned ELISA (OD<0.75), provided the interval between the presumed acute retroviral syndrome and initiating antiretroviral therapy was 6 months or less. Subjects were required to be on a stable antiretroviral regimen and have a CD4+ T cell count >350 cells/mm^3^ and HIV-1 RNA levels <50 copies/ml for at least 6 months. All subjects gave written informed consent, and the study protocol was approved by the AIDS Clinical Trials Group, the NIH Division of AIDS (DAIDS) and the human protection committees of each participating institution.

### Vaccine

The vaccine used in this study was developed by the Vaccine Research Center (VRC), National Institute of Allergy and Infectious Diseases (NIAID), National Institutes of Health (NIH). This vaccine consisted of a 4 plasmid mixture encoding subtype B Gag-Pol-Nef fusion protein and modified envelope constructs from HIV-1 subtypes A, B and C.

### Study Design

This was a randomized, double-blind, placebo-controlled phase I/II clinical trial to assess the safety and immunogenicity of the HIV-1 DNA vaccine in HIV-1-infected subjects who were treated with at least 2 antiretroviral agents during acute or early infection and who maintained an HIV-1 RNA viral load of <50 copies/mL. Five ACTG sites enrolled subjects into this study: Massachusetts General Hospital, Brigham and Women's Hospital, Aaron Diamond AIDS Research Center, University of California at San Diego and University of Washington. The first part of the study (phase 1) was designed to assess the safety and immunogenicity of the vaccine. With 10 active vaccine recipients, there would 80% probability of observing at least one safety endpoint if the per-subject probability is 15%. The second part (phase 2) of the study involved a supervised treatment interruption in order to determine if vaccination with HIVDNA009-00VP resulted in improved immune control of viral replication as evidenced by a reduction in the set-point level of HIV-1 RNA in the absence of antiretroviral therapy.

### Treatment Protocol

#### Phase 1- Therapeutic Vaccination

For the first part of the study, subjects were randomized in a 1∶1 fashion to vaccine (Arm A) versus placebo (Arm B) using permuted blocks. The vaccine or placebo was administered as an intramuscular 1 ml injection using a needle-free Biojector 2000™. Subjects received 4 vaccinations with 4 mg of DNA vaccine or placebo at weeks 0, 4, 8 and 24.

#### Phase 2- Supervised Treatment Interruption

At week 30, all individuals safely completing the therapeutic vaccination phase of the trial were given the opportunity to discontinue therapy. During the supervised treatment interruption phase of this study, subjects were monitored closely and asked to restart antiretroviral therapy if they met the following criteria: a confirmed decline in CD4 count from baseline of >50% or an absolute CD4+ T lymphocyte count of <250 cells/mm^3^, or a confirmed HIV-1 RNA level of >100,000 copies/mL for at least 8 weeks. All subjects were followed until completion of the study at week 52 and those subjects who did not meet criteria to restart therapy or elected not to restart therapy were followed an additional 20 weeks until week 72.

### Outcome Measures

#### Safety

The primary objective of this study was to evaluate the safety of the vaccine. The primary safety endpoint was the development of a grade 3 or higher sign, symptom or laboratory abnormality that was at least possibly related to the vaccine; 2 consecutive viral loads ≥400 copies/mL while on antiretroviral therapy; or 2 consecutive absolute CD4 counts ≤250 cells/mm^3^ while receiving antiviral therapy; or 2 consecutive CD4 counts more than 50% below the baseline CD4 count. Only events that occurred on or subsequent to the first vaccination and within 24 weeks of the last vaccine administration were considered.

#### Immunogenicity

The immunogenicity of VRC-HIVDNA009-00-VP was determined by the following assays: unfractionated interferon-γ ELISPOT, CD4 (CD8-depleted) ELISPOT, CD8 (CD4-depleted) ELISPOT and,lymphocyte proliferation. ELISPOT assays were performed using the following peptide pools (Vaccine Research Center, NIAID, NIH, Bethesda, MD): Gag, Pol-1, Pol-2, Nef, Env-A, Env-B, and Env-C. Baseline ELISPOT responses were determined by taking the geometric mean of pre-entry and entry. Positive responses were defined as a 2-fold increase from baseline that were also ≥100 spot forming cells (SFC) per million PBMC. Since 7 HIV-1 antigens were tested, a positive ELISPOT sum response was defined as a 2-fold increase from baseline that was also ≥700 spot forming cells per million PBMC. Lymphoproliferative responses to CMV, Nef, rt, Gp160, and p24 were assessed by the stimulation index. A stimulation index of ≥5 was considered a positive response.

#### Set-point Viral Load and CD4+ T Cell Count

The main secondary endpoint of this study was the viral load set-point defined as the average of the log_10_ viral load measured at weeks 18, 20 and 22 after antiviral withdrawal (weeks 48, 50, and 52 of the protocol). Similarly, CD4+ T cell counts were determined at the same time points as the viral load measurements. The CD4+ T cell count prior to interruption of therapy was determined by taking the average of the last 2 observations prior to treatment interruption.

#### Study Oversight

The study team conducted a weekly review of adverse events including all reported signs and symptoms and laboratory abnormalities. The study team remained blinded to the randomization assignment of the study subjects. After reviewing all reported events, the team assessed the possible relationship of adverse events to the study vaccine. In addition, an independently appointed Study Monitoring Committee was convened to review the study data, broken down by vaccine and placebo arm.

#### Statistical Analysis

Individuals receiving vaccine versus placebo were summarized using medians and compared using exact Wilcoxon Rank-Sum tests. A Hodges-Lehmann confidence interval was used for the difference in viral load setpoint between arms (vaccine minus placebo). Time-to-event endpoints were compared with exact log-rank tests. All tests were two-sided, 5% level and exploratory.

The viral load set-point analysis was based on subjects who entered the treatment interruption phase of the study and who had an observed viral load set-point. Subjects who restarted antiviral therapy before week 12 of treatment interruption were not included in the viral set-point analysis. A supplemental intent-to-treat sensitivity analysis, which included all subjects, was also performed. In this analysis, the last observed HIV-1 RNA value during treatment interruption was carried forward for any subjects who did not have an observed viral load set-point. Analysis of CD4+ T cell count was also carried out in the same manner.

## Results

### Participant Characteristics and Study Design

Twenty subjects were enrolled in this study (Table 1). Ten subjects received 4 mg vaccine at weeks 0, 4, 8, and 24 (Arm A) and ten subjects received placebo at these same time points (Arm B). Accrual began in May 2004 and closed in April 2006. All subjects were male and the median age was 40 years (39 and 41 years, Arms A and B). The median CD4+ T cell count at baseline was 750 cells/mm^3^ (665 and 934 cells/mm^3^, Arms A and B). All subjects had HIV-1 RNA levels <50 copies/mL at study entry and all received the 4 scheduled vaccinations. At week 30, all 20 subjects elected to discontinue antiretroviral therapy. Visit compliance and data availability were ≥90%.

**Table 1:** Baseline Characteristics by Treatment Arm.

Characteristics	Total (*n* = 20)	Vaccine Arm A (*n* = 10)	Placebo Arm B (*n* = 10)
**Age (Median)**	40	39	41
18–29	1 (5%)	0 (0%)	1 (10%)
30–39	9 (45%)	6 (60%)	3 (30%)
40–49	10 (50%)	4 (40%)	6 (60%)
**Sex**			
Males	20 (100%)	10 (100%)	10 (100%)
**Race/Ethnicity**			
White Non- Hispanic	16 (80%)	9 (90%)	7 (70%)
Hispanic	3 (15%)	0 (0%)	3 (30%)
Asian, Pacific Islander	1 (5%)	1 (10%)	0 (0%)
**IV Drug Use**			
Never	20 (100%)	10 (100%)	10 (100%)
**CD4+ Cells/mm^3^**			
Median	750	665	934
Less than 500	4 (20%)	2 (20%)	2 (20%)
501–750	6 (30%)	5 (50%)	1 (10%)
751–1000	5 (25%)	2 (20%)	3 (30%)
More than 1000	5 (25%)	1 (10%)	4 (40%)

### Vaccine Safety

The vaccine was safe and well tolerated. No subjects experienced grade 3 or 4 signs, symptoms, or laboratory abnormalities that were at least possibly related to the vaccine. Moreover, no subjects exhibited detectable viral loads during the vaccination phase of the study while on antiretroviral therapy. No subject met any primary safety endpoint.

### Treatment Discontinuation

All 20 subjects discontinued antiretroviral therapy at week 30. No subject restarted therapy due to safety endpoints, which included sustained high viral loads or declines of CD4 counts (see Methods). One subject in Arm A elected to re-initiate therapy after 11 weeks of treatment interruption and thus did not contribute to the viral set-point analysis. One subject in Arm B re-initiated therapy after 30 weeks of treatment interruption and did contribute to viral set-point analysis. Thus, 19 subjects were included in the viral load analysis.

**Figure 1:**
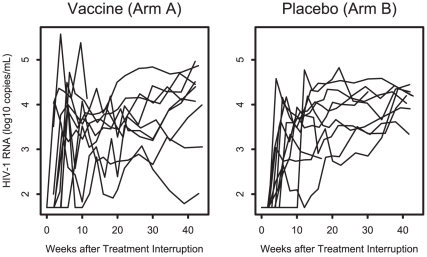
HIV-1 RNA levels following treatment interruption in Arm A (vaccine) and Arm B (placebo). The set-point viral load was determined by measuring HIV-1 RNA between weeks 17–23 of the treatment interruption. The median viral loads at setpoint were 3.5 and 3.7 log_10_ copies/ml in Arms A and B, respectively.

**Figure 2:**
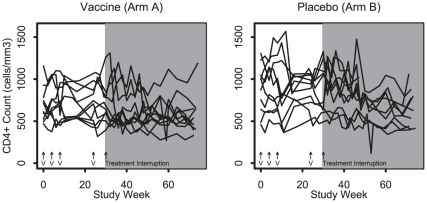
CD4+ T cell counts in Vaccine (Arm A) and Placebo (Arm B). Antiretroviral therapy was discontinued at study week 30. The set-point CD4+ T cell count was determined by measuring T cell subsets between weeks 17–23 of the treatment interruption. The median CD4+ T cell counts at setpoint were 629 and 728 cells/mm^3^ in Arms A and B. The symbol V indicates when vaccine or placebo was administered. The shaded area indicates the time period when subjects were off antiretroviral therapy.

HIV-1 viral loads and CD4 counts are shown in Figure 1 and Figure 2. No significant differences in set-point viral loads were observed between the two groups following treatment discontinuation (median of 3.5 and 3.7 log_10_ RNA copies/ml in Arms A and B, p = 0.50, 95% confidence interval: −0.9 to 0.6 log_10_ copies/ml; sensitivity analysis, n = 20, p = 0.63). There were also no evidence for differences between the two groups in exploratory analyses of peak viral loads during the treatment interruption (median of 4.6 and 4.3 log_10_ copies/ml in Arms A and B, p = 0.10), time to peak viral load (median of 10 vs 17 weeks, p = 0.43), or time to detectable viral load (median of 4 vs 5 weeks, p = 0.34). Moreover, no differences in CD4 counts were observed at set-point following treatment discontinuation (median of 629 and 728 cells/mm^3^ in Arms A and B, p = 0.18). There were also no detectable differences in viral loads or CD4 counts following treatment interruption between the two arms when separately analyzed by initial treatment during acute vs. early infection (data not shown).

All subjects remaining off antiretroviral therapy at week 52 were asked to extend the period of follow-up for an additional 20 weeks (week 72 of the study, week 42 of treatment interruption). Seventeen subjects agreed to be followed for this extended time period. HIV-1 RNA and CD4+ T cell measurements were obtained at week 42 of the treatment interruption phase. The median HIV-1 RNA viral load at week 42 of treatment interruption was 4.4 and 4.2 log_10_ copies/ml for Arms A and B, respectively (p = 0.48). Similarly, CD4+ T cell counts were also determined and were 499 and 698 cells/mm^3^, Arms A and B respectively (p = 0.28)

### Immunogenicity

**Figure 3:**
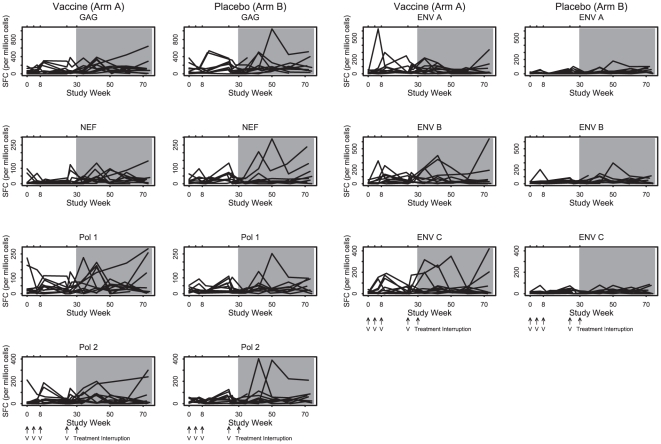
CD4 IFN-γ ELISPOT profile by antigen and treatment arm. Antiretroviral therapy was discontinued at study week 30. Positive responses were defined as a 2-fold increase from baseline that were also ≥100 spot forming cells (SFC) per million PBMC. The symbol V indicates when vaccine or placebo was administered. The shaded area indicates the time period when subjects were off antiretroviral therapy.

**Figure 4:**
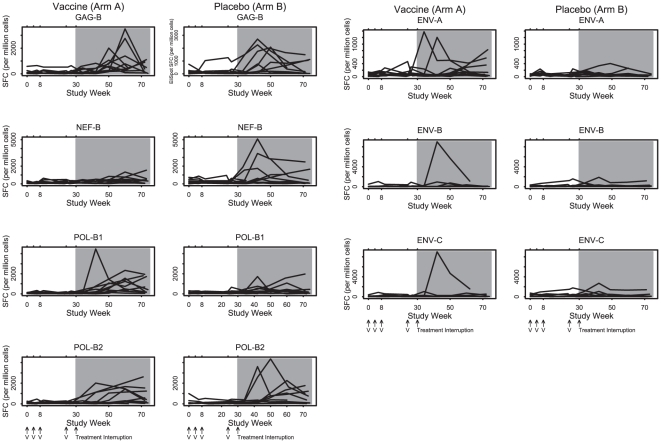
CD8 IFN-γ ELISPOT profile by antigen and treatment arm. Antiretroviral therapy was discontinued at study week 30. Positive responses were defined as a 2-fold increase from baseline that were also ≥100 spot forming cells (SFC) per million PBMC. The symbol V indicates when vaccine or placebo was administered. The shaded area indicates the time period when subjects were off antiretroviral therapy.

**Figure 5:**
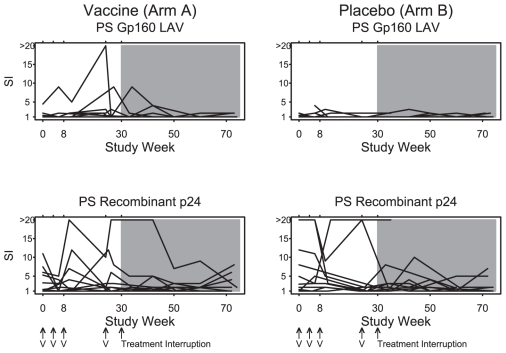
Lymphocyte proliferation responses (Stimulation Index) by antigen and treatment arm. Antiretroviral therapy was discontinued at study week 30. The symbol V indicates when vaccine or placebo was administered. The shaded area indicates the time period when subjects were off antiretroviral therapy.

CD4 and CD8 ELISPOT responses are depicted in Figure 3 and Figure 4. During the vaccination phase (weeks 0 to 30), no evidence for vaccine-augmented ELISPOT responses above baseline responses were observed for any antigen in either group, although a trend of increased Env-specific CD4 ELISPOT responses was noted in Arm A. Modestly increased lymphoproliferative responses to gp160 was observed in Arm A as compared with Arm B (Figure 5; p = 0.03, median 1.3 vs. 1.0 for fold-change increase from baseline to the last two measurements before treatment interruption). Following the treatment interruption at week 30, augmented CD8 ELISPOT responses were observed to all antigens in response to active virus replication. However, no differences in ELISPOT responses were observed between the two groups following withdrawal of antiretroviral therapy.

## Discussion

ACTG A5187 assessed the safety, immunogenicity and viral load set-point following discontinuation of antiretroviral therapy in HIV-1-infected subjects who received either HIV-1 DNA vaccine or placebo. All subjects enrolled in the study were healthy HIV-1-infected subjects who were diagnosed and treated with antiretroviral therapy during acute or early HIV-1 infection. Twenty subjects were followed through phase 1 (the immunization phase) of the study and entered phase 2 (the treatment interruption) of the study. Of the 20 subjects who discontinued antiretroviral therapy, 2 subjects restarted therapy and 18 remained off therapy at the end of the study. This study was designed using a relatively small sample size based on limited availability of vaccine and anticipated difficulty in recruitment of subjects with treated acute or early HIV-1 infection.

The primary objective of this study was to determine the safety of the HIV-1 DNA vaccine when used therapeutically in healthy HIV-1-infected subjects. In this study, no subject experienced any serious (grade 3 or 4) adverse events during the administration of the HIV-1 DNA vaccine, and the vaccine was safe and well tolerated. These safety data are consistent with a prior study utilizing this vaccine in HIV-1-uninfected individuals[2].

All subjects safely discontinued antiretroviral therapy at week 30, and no safety endpoints were reached during this phase of the trial. No subjects met predetermined criteria to restart therapy. These findings contrast with those reported in the SMART study that assessed the efficacy of continuous versus episodic use of antiretroviral therapy in persons with chronic HIV-1 infection[7]. Results from the SMART study suggested that episodic use of antiretroviral therapy resulted in a significantly increased risk of opportunistic infections and death compared to persons taking continuous antiviral therapy[7]. In ACTG A5187, antiretroviral therapy was similarly discontinued although there were differences between the treatment interruption strategies employed in ACTG A5187 compared to the SMART study. The main differences were that subjects enrolled in ACTG A5187 were treated during acute or early HIV-1 infection compared to subjects enrolled in the SMART study who were treated during the chronic phase of infection. Subjects enrolled in ACTG A5187 were therefore likely healthier with more intact immune systems then subjects enrolled in the SMART study[4], [5]. Moreover, the method of treatment discontinuation was different in ACTG A5187 compared to the SMART study. In the present study, individuals underwent a “terminal interruption” as opposed to the episodic interruption guided by CD4+ T cell counts used in the SMART study and also had more frequent CD4 and HIV-1 RNA measurements. It is important to establish the safety of discontinuing treatment in individuals who begin therapy during acute or early HIV-1 infection, since the clinical benefit of early treatment has not yet been demonstrated. The present study therefore begins to provide safety data for treatment interruption in this population, although the results are limited by the small number of subjects studied.

Although the HIV-1 DNA vaccine was safe and well tolerated, the vaccine exhibited minimal immunogenicity that appeared lower than what has been reported in prior studies in HIV-1-uninfected volunteers in which significant humoral and cellular immune responses were observed[2]. It is not clear why this vaccine was not as immunogenic in HIV-1-infected individuals. One possible explanation is that the subjects in this clinical trial had pre-existing HIV-1-specific immune responses at baseline and that the HIV-1 DNA vaccine may not have been able to boost these responses above that baseline. Given the small size and limited power of this study, only large effects on immunogenicity would be detected. Therefore, it is possible that more modest immunogenicity was not detected due to the power of the study. Although all study subjects had relatively healthy CD4 counts, it is notable that the placebo group had higher baseline CD4 counts then the vaccine recipients although this was not a statistically significant difference.

One notable finding of this study is the low viral load set-point in subjects who completed the treatment interruption phase of the study. Although there were no evidence for vaccine efficacy in terms of a difference in viral set-points between subjects receiving the vaccine compared to the placebo (p = 0.50), both the vaccine group and the placebo group had low median viral set-points of 3.5 and 3.7 log _10_ respectively. When these viral load set-points are compared to those described in the natural history Multicenter AIDS Cohort (MACS) study[8], the set-points appear markedly lower. In the MACS cohort, the average viral load in untreated subjects approximately 12 months following seroconversion was 4.45 log_10_ (28,240 RNA copies/ml)[8] compared to an average viral load of 3.6 log _10_ (4,000 RNA copies/ml) in subjects completing the treatment interruption phase of the present study. Since there was not a significant difference between the vaccine group and placebo group, it is unlikely that administration of the HIV-1 DNA vaccine contributed to the low viral load set-point in this study. It is therefore possible that early treatment during acute or early HIV-1 infection may have resulted in better control of viral replication once antiretroviral therapy was discontinued, although this hypothesis needs to be evaluated in larger prospective studies. Such a finding would be consistent with previously described observations[5], [9], although the durability of control following treatment interruption may be limited[10]. Subjects that were observed over a long period of time (through week 72 of the study), had higher levels of viremia that trended towards levels reported in the MACS cohort[8]. These findings suggest a possible role of antiretroviral therapy in persons with acute and early HIV-1 infection and indicate that further studies should be performed to determine if such therapy is beneficial. Therapeutic vaccine studies utilizing more potent HIV-1 vaccine candidates should also be considered.

## Supporting Information

Protocol S1 Trial Protocol.
(0.60 MB DOC)

Checklist S1 Consort Checklist.
(0.19 MB DOC)
