# Supplementary material for: Safety and Immunogenicity of Therapeutic DNA Vaccination in Individuals Treated with Antiretroviral Therapy during Acute/Early HIV-1 Infection
Source: PLoS One. 2010 May 10;5(5):e10555. doi: 10.1371/journal.pone.0010555 (PMC2866663; doi:10.1371/journal.pone.0010555)
Supplement: Protocol S1 — Trial Protocol. (0.60 MB DOC) [file pone.0010555.s001.doc]

**A5187**

**A PHASE I/II CLINICAL TRIAL TO EVALUATE THE SAFETY AND IMMUNOGENICITY OF THE HIV-1 DNA VACCINE VRC-HIVDNA009-00-VP (GAG-POL-NEF-MULTICLADE ENV) IN HIV-1-INFECTED SUBJECTS TREATED DURING ACUTE INFECTION**

**A Limited CenterTrial of the Adult AIDS Clinical Trials Group (AACTG)**

**Sponsored by:**

**The National Institute of Allergy**

**and Infectious Diseases**

**Vaccine Provided by:**

**Vaccine Research Center**

**National Institutes of Health**

**IND # ________**

The AACTG Immunology Research Agenda Committee (IRAC):

**Alan Landay, Ph.D., Chair**

**Protocol Co-Chairs: Dan H. Barouch, M.D., Ph.D.**

**Eric S. Rosenberg, M.D.**

**Vice Chair: Daniel R. Kuritzkes, M.D.**

DAIDS Clinical Representative: Elizabeth Adams, M.D.

**Clinical Trials Specialist: Vicki Stocker, R.N., M.S.**

**FINAL Version 1.0**

**September 5, 2003**

TABLE OF CONTENTS

Page

SITES PARTICIPATING IN THE STUDY [4](#__RefHeading___Toc48966213)

PROTOCOL TEAM ROSTER [5](#__RefHeading___Toc48966214)

STUDY MANAGEMENT [9](#__RefHeading___Toc48966216)

SCHEMA [11](#__RefHeading___Toc48966217)

1.0 STUDY OBJECTIVES [13](#__RefHeading___Toc48966219)

1.1 Primary Objective [13](#__RefHeading___Toc48966220)

1.2 Secondary Objectives [13](#__RefHeading___Toc48966221)

2.0 INTRODUCTION [13](#__RefHeading___Toc48966222)

2.1 Background [13](#__RefHeading___Toc48966223)

2.2 Rationale [17](#__RefHeading___Toc48966224)

3.0 STUDY DESIGN [18](#__RefHeading___Toc48966225)

3.1 Study Overview [18](#__RefHeading___Toc48966226)

3.2 Criteria to Restart HAART [19](#__RefHeading___Toc48966227)

4.0 SELECTION AND ENROLLMENT OF SUBJECTS [20](#__RefHeading___Toc48966228)

4.1 Inclusion Criteria [20](#__RefHeading___Toc48966229)

4.2 Exclusion Criteria [22](#__RefHeading___Toc48966230)

4.3 Study Enrollment Procedures [23](#__RefHeading___Toc48966231)

5.0 STUDY TREATMENT [24](#__RefHeading___Toc48966232)

5.1 Regimen, Administration, and Duration [24](#__RefHeading___Toc48966233)

5.2 Product Formulation and Preparation [25](#__RefHeading___Toc48966234)

5.3 Product Supply, Distribution, and Pharmacy [26](#__RefHeading___Toc48966235)

5.4 Concomitant Medications [26](#__RefHeading___Toc48966236)

6.0 CLINICAL AND LABORATORY EVALUATIONS [28](#__RefHeading___Toc48966237)

6.1 Schedule of Events: Part 1 [28](#__RefHeading___Toc48966238)

6.2 Definitions for Schedule of Events – Timing of Evaluations [30](#__RefHeading___Toc48966239)

6.3 Special Instructions and Definitions of Evaluations [31](#__RefHeading___Toc48966240)

6.4 Off-Vaccine Requirements [37](#__RefHeading___Toc48966241)

7.0 TOXICITY MANAGEMENT [38](#__RefHeading___Toc48966242)

8.0 CRITERIA FOR DISCONTINUATIONOF VACCINE [39](#__RefHeading___Toc48966243)

9.0 STATISTICAL CONSIDERATIONS [40](#__RefHeading___Toc48966244)

9.1 General Design Issues [40](#__RefHeading___Toc48966245)

9.2 Endpoints [40](#__RefHeading___Toc48966246)

9.3 Sample Size and Accrual [41](#__RefHeading___Toc48966247)

9.4 Monitoring [43](#__RefHeading___Toc48966248)

9.5 Analyses [43](#__RefHeading___Toc48966249)

10.0 DATA COLLECTION AND MONITORING AND ADVERSE EXPERIENCE REPORTING [44](#__RefHeading___Toc48966250)

10.1 Records to Be Kept [44](#__RefHeading___Toc48966251)

10.2 Role of Data Management [44](#__RefHeading___Toc48966252)

10.3 Clinical Site Monitoring and Record Availability [45](#__RefHeading___Toc48966253)

10.4 Serious Adverse Experience (SAE) Reporting [45](#__RefHeading___Toc48966254)

11.0 HUMAN SUBJECTS [45](#__RefHeading___Toc48966255)

11.1 Institutional Review Board (IRB) Review and Informed Consent [45](#__RefHeading___Toc48966256)

11.2 Subject Confidentiality [46](#__RefHeading___Toc48966257)

11.3 Study Discontinuation [46](#__RefHeading___Toc48966258)

12.0 PUBLICATION OF RESEARCH FINDINGS [46](#__RefHeading___Toc48966259)

13.0 BIOHAZARD CONTAINMENT [46](#__RefHeading___Toc48966260)

14.0 REFERENCES [47](#__RefHeading___Toc48966261)

APPENDIX I Definition of Antiretroviral Syndrome [50](#__RefHeading___Toc48966263)

APPENDIX IISpecimen Collection, Processing, Storage, and Shipment [51](#__RefHeading___Toc48966264)

APPENDIX III Sample Informed Consent 55

###### SITES PARTICIPATING IN THE STUDY

A5187 is a limited site study. Adult AIDS Clinical Trials Units (AACTUs) eligible to participate are:

- Site 0101, Harvard (Massachusetts General Hospital)
- Site 0107, Brigham and Women’s Hospital

###### PROTOCOL TEAM ROSTER

Protocol Co-Chair

Dan H.Barouch, M.D., Ph.D.

Instructor in Medicine

Beth Israel Deaconess Medical Center

Division of Viral Pathogenesis

Research East 117

330 Brookline Avenue

Boston, MA 02215

Phone: (617)-667-4434

FAX: (617)-667-8210

E-mail: dbarouch@bidmc.harvard.edu

Protocol Co-Chair and Immunologist

Eric S. Rosenberg, M.D.

Assistant Professor of Medicine

Massachusetts General Hospital

Division of Infectious Diseases

GRJ 529A

55 Fruit Street

Boston, MA 02114

Phone: (617)-724-7519

FAX: (617)-726-7416

E-mail: erosenberg1@partners.org

Vice Chair

Daniel R. Kuritzkes, M.D.

Associate Professor of Medicine

Director of AIDS Research

Brigham and Women’s Hospital

Partners AIDS Research Center

65 Landsdowne Street, Room 449

Cambridge, MA 02139

Phone: (617)-768-8371

FAX: (617)-768-8738

E-mail: dkuritzkes@partners.org

DAIDS Clinical Representative

Elizabeth Adams, M.D.

TRP, HIVRB

DAIDS, NIAID, NIH,

Room 5216

6700-B Rockledge Drive, MSC 7624

Bethesda, MD 20892-7624

Phone: (301) 496-0700

FAX: (301) 435-9282

E-mail: [eadams@niaid.nih.gov](mailto:eadams@niaid.nih.gov)

Clinical Trials Specialist

Vicki Stocker, R.N., M.S.

AACTG Operations Center

8757 Georgia Avenue, 12th Floor

Silver Spring, MD 20910-3714

Phone: (301) 628-3313

FAX: (301) 628-3302

E-mail: vstocker@s-3.com

## Statisticians

Ronald J. Bosch, Ph.D.

Section Head, Immunology

Statistical and Data Analysis Center

Harvard School of Public Health

FXB Building, Room 603

651 Huntington Avenue

Boston, MA 02115-6017

Phone: (617) 432-3024

FAX: (617) 432-2843

E-mail: ronbosch@sdac.harvard.edu

Statisticians (cont.)

Evgenia Aga, M.Sc.

Biostatistician

Statistical Data and Analysis Center

Harvard School of Public Health

Center for Biostatistics in AIDS Research

FXB Building, Room 504

651 Huntington Avenue

Boston, MA 02115

Phone: (617) 432-3029

FAX: (617) 432-3163

E-mail: eaga@sdac.harvard.edu

RAC Representative

Gail Skowron, M.D.

Roger Williams Medical Center

Division of Infectious Diseases

825 Chalkstone Avenue

Providence, RI 02908-4735

Office Phone: (401) 456-2437

Conference Phone: (401) 456-2591

FAX: (401) 456-6839

E-mail: gail_skowron@brown.edu

Data Manager

Laurie S. Myers, M.S.

Frontier Science & Technology Research Foundation Inc.

4033 Maple Road

Amherst, NY 14226-1056

Phone: (716) 834-0900 x338

FAX: (716) 834-8432

E-mail: myers.laurie@fstrf.org

DAIDS Pharmacist

Ana Martinez, R.Ph.

Chief, Pharmaceutical Affairs Branch

DAIDS, NIAID, NIH

Room 5115

6700-B Rockledge Drive, MSC-7620

Bethesda, MD 20892-7620

Phone: (301) 435-3734

FAX: (301) 402-1506

E-mail: [am30c@nih.gov](mailto:am30c@nih.gov)

Field Representative

Teri Flynn, R.N., A.N.P.
Harvard/BMC ACTG Coordinator
Massachusetts General Hospital
55 Fruit Street
Founders Bldg., 8th Floor
Boston, MA  02114
Phone: (617) 726-3819
FAX: (617) 742-4795
E-mail: tflynn@partners.org

Laboratory Technologist

David Shugarts, M.A.

Laboratory Manager

University Hospital Administration Building

4210 East 11th Avenue**,** Room 283

Denver, CO 80262

Phone: (303) 315-1827

FAX: (303) 315-1816
E-mail: david.shugarts@uchsc.edu

CCG Representatives

Rolf Christensen, D.D.S., M.H.A.

CAB Member/University of Washington

P.O. Box 95730

Seattle, WA 98145-2730

Phone: (206) 543-6501

FAX: (206) 634-9112

E-mail: rolfsea@msn.com

Robert Irizarry, B.B.A.

CAB Member

University of Puerto Rico School of Medicine

Adult AIDS Clinical Trials Unit (ACTU Project)

Infectious Diseases Section

Medicla Sciences Campus

P.O. Box 365067

San Juan, PR 00936-0408

Phone: (787) 767-9193

FAX: (787) 754-8002

E-mail: irizarry00936@yahoo.com

Vaccine Research Center Representatives

Richard A. Koup, M.D.

Director of Human Immunology

National Institutes of Health

Vaccine Research Center

Building 40, Room 3502

40 Convent Drive

Bethesda, MD 20892

Phone: (301) 594-8574

FAX: (301) 480-2779

E-mail: rkoup@mail.nih.gov

Barney S.Graham, M.D., Ph.D.

Director of Clinical Studies

National Institutes of Health

Vaccine Research Center

Bldg. 40, Rm 2502

40 Convent Drive, MSC 3017

Bethesda, MD 20892-3017

Phone: (301) 594-8468

FAX: (301) 480-2771

E-mail: bgraham@nih.gov

Gary Nabel, M.D., Ph.D.

Director, Vaccine Research Center

National Institutes of Health

Building 40, Room 4502

40 Convent Drive

Bethesda, MD 20892

Phone: (301) 496-1852

FAX: (301) 480-0274

E-mail: gnabel@mail.nih.gov

Vaccine Research Center Representatives (cont.)

Phillip L. Gomez III, Ph.D.

Director of Vaccine Production

National Institutes of Health

Vaccine Research Center

Building 40, Room 5504

40 Convent Drive

Bethesda, MD 20892-3011

Phone: (301) 594-8485

FAX: (301) 480-2788

E-mail: pgomez@mail.nih.gov

Laboratory Data Coordinator

Amanda Zadzilka

Frontier Science & Technology Research Foundation Inc.

4033 Maple Road

Amherst, NY 14226-1056

Phone: (716) 834-0900x282

FAX: (716) 833-0655

E-mail: [zadzilka.amanda@fstrf.org](mailto:zadzilka.amanda@fstrf.org)

###### STUDY MANAGEMENT

All questions concerning this protocol should be sent via e-mail to [actg.teamA5187@fstrf.org](mailto:actg.teamA5187@fstrf.org). The appropriate team member will respond via e-mail with a "cc" to [actg.teamA5187@fstrf.org](mailto:actg.teamA5187@fstrf.org). A response should generally be received within 24 hours (Monday-Friday).

Protocol e-mail group: Sites registering to this study should contact the Computer Support Group at the Data Management Center via e-mail (actg.user.support@fstrf.org) to have the relevant personnel at the site added to the actg.protA5187 e-mail group as soon as possible. Inclusion in the protocol e-mail group will ensure that sites receive important information about the study during its implementation and conduct.

FOR QUESTIONS CONCERNING CLINICAL MEDICAL MANAGEMENT, INCLUDING ENTRY CRITERIA, TOXICITY MANAGEMENT, CONCOMITANT MEDICATIONS, AND CO-ENROLLMENT, THE PROTOCOL CO-CHAIRS/PROTOCOL VICE CHAIR WILL RESPOND.

- Send an e-mail message to [actg.teamA5187@fstrf.org](mailto:actg.teamA5187@fstrf.org) (ATTN: Dan Barouch, Eric Rosenberg, and Daniel Kuritzkes).
- Include the protocolnumber, patient identification number(PID), and a brief relevant history.

FOR NONCLINICAL QUESTIONS ABOUT INCLUSION/EXCLUSION CRITERIA, THE CRF SCHEDULE OF EVENTS, CASE REPORT FORMS, RANDOMIZATION, TRANSFERS, DELINQUENCIES, AND OTHER DATA MANAGEMENT ISSUES, THE DATA MANAGER WILL RESPOND:

- Send an e-mail message to [actg.teamA5187@fstrf.org](mailto:actg.teamA5187@fstrf.org) (ATTN: Laurie Myers).
- Include the protocol number, PID, and a detailed question.

FOR RANDOMIZATION QUESTIONS OR PROBLEMS AND SID LISTS, THE SDAC/DMC PROGRAMMERS WILL RESPOND:

- Call the SDAC/DMC Randomization Desk at (716) 834-0900, x301 or e-mail sdac.random.desk@fstrf.org.

FOR COMPUTER AND SCREEN PROBLEMS, THE SDAC/DMC PROGRAMMERS WILL RESPOND:

- Send an e-mail message to actg.user.support@fstrf.org.

STUDY MANAGEMENT (Cont.)

FOR PROTOCOL QUESTIONS, THE CLINICAL TRIALS SPECIALIST WILL RESPOND:

- Send an e-mail message to actg.teamA5187@fstrf.org (ATTN: Vicki Stocker).

FOR REQUESTS FOR COPIES OF THE PROTOCOL:

- Hard copies: Send an e-mail message to ACTG.OPS@fstrf.org (ATTN: Diane Delgado).
- Electronic copies can be downloaded from the Members area of the AACTG Web site ([http://aactg.s-3.com](http://aactg.s-3.com/)).

FOR PROTOCOL REGISTRATION:

- Send an e-mail message to actg.protreg@fstrf.org.

FOR QUESTIONS OR PROBLEMS REGARDING STUDY VACCINE PRODUCT, DOSE, SUPPLIES, RECORDS, AND RETURNS:

- Call (Ana Martinez), Protocol Pharmacist, at (301) 496-8213.

FOR IND #:

- Call the DAIDS RegulatoryCompliance Center at (301) 564-6400.

TO ORDER STUDY DRUG:

- Call the Clinical Research Products Management Center at (301) 294-0741.

FOR SERIOUS ADVERSE EXPERIENCES (SAE) QUESTIONS:

Contact the DAIDS Regulatory Compliance Center SAE Office

- Send an e-mail message to actg.adr@fstrf.org, or
- Call 1-800-537-9979.

Any phone calls must be documented by e-mail to [**actg.teamA5187@fstrg.org**](mailto:actg.teamA5187@fstrg.org). This will be the site’s responsibility.

Additional information concerning study management of AACTG studies can be found on the AACTG WEB page ([*http://aactg.s-3.com*](http://aactg.s-3.com/)).

###### SCHEMA

A PHASE I**/**II CLINICAL TRIAL TO EVALUATE THE SAFETY AND IMMUNOGENICITY OF THE HIV-1 DNA VACCINE VRC-HIVDNA009-00-VP (GAG-POL-NEF-MULTICLADE ENV) IN HIV-1-INFECTED SUBJECTS TREATED DURING ACUTE INFECTION

DESIGN: A5187 is a randomized, double-blind, placebo-controlled phase I**/**II clinical trial to assess the safety and immunogenicity of the HIV-1 DNA vaccine VRC-HIVDNA009-00-VP (Gag-Pol-Nef-multiclade Env) in HIV-1-infected subjects who were treated with highly active antiretroviral therapy(HAART), defined as at least 2 antiretroviral agents, during acute infection and maintaining a viral load suppression of < 50 copies/mL.The first part of the study will assess the safety and immunogenicity of VRC-HIVDNA009-00VP. The second part of the study will involve a supervised treatment interruption (STI) in order to obtain preliminary exploratory data to assess whether DNA vaccination results in improved immune control of viral replication as evidenced by a reduction in the setpoint level of HIV-1 RNA in the absence of antiretroviral therapy.

DURATION: 72 weeksfor each subject.

SAMPLE SIZE: 20 subjects, 10 per arm.

POPULATION: 20 healthy HIV-1-infected adult subjects aged 18-50 treated with HAART during acute HIV-1 infection on stable antiretroviral regimens with CD4+ T-cell counts >350 cells/mm3 and HIV-1 RNA levels <50 copies/mL for at least 6 months.

REGIMEN:

Part 1 – Therapeutic Vaccination (Weeks 0-30)

| Arm | N | Vaccine  (4 mg/mL) | Placebo  (Phosphate-buffered saline [PBS]) | Week 0 | Week 4 | Week 8 | Week 24 |
| --- | --- | --- | --- | --- | --- | --- | --- |
| A | 10 | 1 mL | - | x | x | x | x |
| B | 10 | - | 1 mL | x | x | x | x |
|  |  |  |  |  |  |  |  |
| Total | 20 |  |  |  |  |  |  |

###### SCHEMA (cont.)

Part 2 – Supervised Treatment Discontinuation (Weeks 30-72)

| Week 30 | Antiretroviral treatment discontinued in subjectswho do not reach a safety endpoint. |
| --- | --- |
| Week 30-52 | Antiretroviral treatment held in subjects who continue to meet criteria |
| Week 52**-**72 | Antiretroviral treatment resumed in subjects at the discretion of the subject’s primary physician based on current clinical guidelines. |

# STUDY OBJECTIVES

## 1.1 Primary Objective

To evaluate the safety of VRC-HIVDNA009-00-VP in healthy HIV-1-infected subjects on stable antiretroviral regimens.

## 1.2 Secondary Objectives

1.2.1 To evaluate the immunogenicity of VRC-HIVDNA009-00-VP in healthy HIV-1-infected subjects as determined by ELISPOT and intracellular cytokine staining (ICS) assays.

1.2.2 To obtain preliminary exploratory data to assess whether DNA vaccination results in improved control of viral replication as evidenced by a lower setpoint viral RNA following a supervised discontinuation of antiretroviral therapy.

# INTRODUCTION

## 2.1 Background

Over 30 million individuals worldwide are infected with HIV-1, the etiologic agent of AIDS. In the United States and Western industrialized world, the development of highly active antiretroviral therapy (HAART) has led to substantial declines in morbidity and mortality (1). However, HAART is associated with high costs, poor compliance, significant toxicities, and the emergence of drug-resistant viruses. More importantly, over 90% of HIV-1-infected individuals live in developing countries and have little or no access to HAART. The need for a safe and effective therapeutic vaccine to improve immune control of viral replication and reduce the need for antiretroviral medications in HIV-1-infected individuals is therefore paramount.

### *Immune Control of HIV-1 Replication*

The importance of CD8+ cytotoxic T lymphocytes (CTL) in containing HIV-1 replication was appreciated soon after the onset of the AIDS epidemic. A temporal correlation was observed between the decline of primary viremia in HIV-1-infected humans and the appearance of virus-specific CD8+ CTL. Major expansions of CD8+ CTL were detected during primary viremia at a time when serum-neutralizing antibody (NAb) activity was notably absent (2-4). In a prospective longitudinal study, virus-specific CTL responses were correlated with the control of chronic HIV-1 replication (5). Studies using tetramer staining technology also demonstrated that HIV-1-specific CTL responses in chronically infected individuals were present at high frequencies, with CD8+ T cells specific for a single epitope often comprising over 1% of circulating CD8+ T cells (6, 7). Moreover, the importance of CD8+ CTLs in controlling SIV replication in rhesus monkeys has been directly established by studies in which *in vivo* depletion of CD8+ T cells resulted in a rapid and dramatic increase in plasma viremia (8). These data demonstrate that CD8+ CTLs play a critical role in the control of HIV-1 replication.

The importance of CD4+ helper T lymphocyte responses in controlling HIV-1 replication has also been established. In contrast to robust CTL responses, HIV-1-specific CD4+ T lymphocyte responses are typically weak or absent in most chronically HIV-1-infected individuals. However, effective treatment with HAART in acutely infected individuals has been shown to preserve and augment HIV-1-specific CD4+ T cell responses (9, 10). Supervised treatment interruptions (STIs) in these patients were then shown to augment virus-specific immune responses and improve immune control of viral replication following the withdrawal of HAART (11). Importantly, a subset of these patients have maintained effective immune control of viral replication for a prolonged period of time without HAART. Similar results have also been reported in studies in SIV-infected rhesus monkeys in which STIs augmented immune responses and provided improved control of viral replication following the discontinuation of therapy (12). It has been hypothesized that the preservation of virus-specific CD4+ T lymphocytes in patients treated during acute HIV-1 infection provides the immunologic help necessary for expanding and maintaining HIV-1-specific CD8+ CTLs (11, 13).

*Preclinical DNA Vaccine Trials*

Plasmid DNA vaccination involves the administration of purified plasmid DNA encoding an antigen. Plasmid DNA is typically injected into skeletal muscle or, alternatively, inoculated as plasmid-coated beads by gene gun into the epidermis. The protein is expressed in transfected mammalian cells, including macrophages and dendritic cells, enters into both the MHC class I and class II processing pathways, and elicits strong and persistent humoral and cellular immune responses (14-16). Although DNA vaccination has not prevented infection with AIDS viruses in animal models, recent reports have demonstrated that immune responses elicited by plasmid DNA vaccines were capable of controlling viral replication and preventing disease progression following pathogenic viral challenges in rhesus monkeys (17-19). Although CTL-based vaccines may be limited by the late development of viral mutations that result in viral escape from CTL recognition (20), plasmid DNA vaccines represent a promising strategy for the development of prophylactic and therapeutic HIV-1 vaccines. Plasmid DNA vaccines have also been combined with other live vectors in effective combination prime-boost vaccination strategies (19, 21).

*VRC Protocol 004*

The Vaccine Research Center (VRC) is currently conducting a phase I clinical trial, VRC Protocol 004, with the DNA vaccine VRC-HIVDNA009-00-VP that will be utilized in this study. Pre-clinical animal studies demonstrated a lack of toxicity and no induction of autoimmunity to intramuscular injections of the DNA vaccine. VRC 004 is testing the safety, tolerability, and immunogenicity of VRC-HIVDNA009-00-VP in HIV-1-uninfected human volunteers at doses of 2 mg, 4 mg, and 8 mg. VRC-HIVDNA009-00-VP is composed of 4 DNA plasmids in a 3:1:1:1 ratio designed to express a Gag-Pol-Nef fusion protein from clade B and Env from clades A, B, and C, respectively. This study opened to accrual in November 2002. The study is designed to enroll 50 healthy volunteers into three sequential groups as follows: Group 1: randomize 7 subjects (5:2 ratio) to receive three vaccinations with 2 mg study agent or placebo; Group 2: randomize 7 subjects (5:2 ratio) to receive three vaccinations with 4 mg study agent or placebo; Group 3: randomize 36 subjects to Group 3a (18 subjects in a 15:3 ratio receive three vaccinations with 4 mg study agent or placebo) or Group 3b (18 subjects in a 15:3 ratio receive three vaccinations with 8 mg study agent or placebo). All subjects receive injections administered at study weeks 0, 4, and 8.ACTG A5187 will use the 4**-**mg dose of VRC-HIVDNA009-00-VP that has been well-tolerated to date in VRC 004.

As of June 26, 2003, there were 33 subjects enrolled in VRC 004. Blinded, interim study results indicate that the injections have been well tolerated to date. The three vaccination series (administered at week 0, week 4, and week 8) have been completed in 7 of 7 subjects randomized to 2 mg/placebo, 13 of 16 subjects randomized to 4 mg/placebo, and 6 of 10 subjects randomized to 8 mg/placebo. One subject with Gilbert’s syndrome was discontinued from the vaccination schedule due to an asymptomatic grade 3 bilirubin (possibly related to study agent). Two other grade 3 adverse events have occurred: one muscle strain (unrelated) and one asymptomatic neutropenia (possibly related). Two grade 4 adverse events have occurred: one acute hepatitis B infection (unrelated) and one HIV infection (unrelated). Among the 29 subjects who have completed one or more 7-day diary cards, the self-assessed reactogenicity has generally been none or mild; 7 of 29 reported at least one moderate local symptom, and 4 of 29 reported at least one moderate systemic symptom. All subjects are continuing with their study visits.

*Other**Clinical DNA Vaccine Trials*

The first clinical trial of a DNA vaccine in HIV-infected individuals evaluated intramuscular injection of a DNA vaccine produced by Apollon expressing the Env and Rev proteins. This study provided extensive safety data and revealed no significant clinical or laboratory findings in any of the subjects. In particular, there was no evidence of antinuclear antibody formation, muscle enzyme elevation, or changes in blood pressure and pulse (22). AIDS Vaccine Evaluation Group Protocol 031 (AVEG 031) investigators also found that immunization with an HIV-1 DNA vaccine-expressing Gag-Pol, also produced by Apollon, elicited HIV-1-specific T lymphocyte responses in HIV-uninfected volunteers.

Results of DNA vaccine clinical trials for other infectious diseases have also been reported. A phase I study of a malaria gene-based DNA vaccine demonstrated that CTLs and IFN-γ-producing T lymphocytes could be induced in naïve human volunteers (23). In a phase I trial in 20 healthy adult volunteers to test the safety, tolerability, and immune responses to a DNA vaccine for malaria (containing a backbone very similar to that being proposed in the current study) there were no severe or serious adverse events and no significant clinical or laboratory findings (24). Anti-dsDNA antibodies were not detected. Furthermore, CTLs reactive with the expressed *Pf*CSP protein were observed, although no antigen-specific antibodies were detected. In a phase I study of a DNA vaccine encoding hepatitis B surface antigen, the vaccine was delivered by the gold bead particle acceleration PowderJect XR1 gene gun into six hepatitis B seronegative subjectsand one seropositive subject. Although none of the seronegative subjects developed primary immune responses in the form of antibodies, the vaccine was well tolerated with minimal, mostly local reactions (25).

*Design Concept of a Multivalent Recombinant HIV-1 DNA Vaccine*

The use of multiple gene products in an HIV vaccine may increase the epitopic breadth of the cellular immune response. A recent study has demonstrated that viral escape from a single immunodominant CTL epitope can result in loss of immune control of viremia and progressive clinical disease in rhesus monkeys (20). This suggests that it will be important for vaccines to generate CTL responses against multiple epitopes. Moreover, there are at least 9 major subtypes (A, B, C, D, F, G, H, J, and K) and dozens of recombinant forms of HIV-1 (26). While the immunological relevance of HIV genetic diversity is not fully understood (27, 28), the use of multivalent vaccines, containing a defined mixture of immunogens from a number of prevalent subtypes might be a feasible approach to achieve broadly protective HIV vaccines. This approach is the foundation for the design of VRC-HIVDNA009-00-VP, which incorporates HIV genes (*gag,* *pol, nef*) from Clade B as well as more diverse *env* genes from Clades A, B, and C. For a description of the design and production of the investigational vaccine that will be used in A5187, VRC-HIVDNA009-00-VP, please refer to the most recent investigator's brochure.

## 2.2 Rationale

*Hypothesis*

This study will test the hypothesis that the DNA vaccine VRC-HIVDNA009-00-VP is safe in subjects who initiated antiretroviral therapy during acute HIV-1-infection. This study will also obtain preliminary information regarding immunogenicity and whether DNA vaccination results in improved immune control of viral replication following treatment discontinuation.

*Study Rationale*

In the STI trials involving patients who were treated during acute HIV-1 infection, the improved immune control of viral replication with subsequent treatment interruptions was likely due to augmented virus-specific immune responses stimulated by re-exposure to autologous virus (11). Therapeutic vaccination of HIV-1-infected individuals offers a potential strategy to augment virus-specific immune responses that may be safer than STIs and does not involve a burst of autologous virus replication. Moreover, patients with treated acute HIV-1 infection may be a particularly promising patient population in which to study the potential beneficial effects oftherapeutic vaccination since they have a relatively preserved immune system and have been shown to benefit from STIs (10, 11). Whether therapeutic vaccination will have beneficial effects comparable with STIs, however, is not yet known, although a preliminary study in rhesus monkeys using recombinant a poxvirus-based vaccine suggests the feasibility of this approach (29).

The immunogenicity of DNA vaccines in nonhuman primate models (17) provides a strong rationale for investigating plasmid DNA vaccines as therapeutic vaccines in humans. In this phase I**/**II clinical trial, we will study the safety and immunogenicity of a plasmid DNA vaccine in patients treated during acute HIV-1 infection. In addition, we will obtain preliminary exploratory data regarding the ability of the vaccine to reduce setpoint viral RNA following a supervised discontinuation of therapy. The correlates of immune protection are largely unknown and although in-vitro studies will allow us to assess HIV-specific immune responses, the best way to test the functionality of immune response is to discontinue therapy and investigate the ability for host immune responses to control viral replication. We will be following anamnestic immune responses in addition to viral load during the STI. Accumulating evidence in the literature suggests that the quality of immune responses is critically important and is not fully captured by the IFNg ELISPOT or ICS responses. Assessing secondary responses following re-exposure to virus is therefore an important component of assessing vaccine immunogenicity, and provides further justification for the STI. Since there are no clear clinical guidelines for how long treatment should be continued once initiated duringacute infection and no evidence that continued therapy is beneficial in this setting, it would be difficult to argue that these individuals need to remain on therapy. From this perspective, incorporating an STI into this study would achieve two goals: it provides the patients with an opportunity to stop treatment in an organized and monitored setting, and it might provide preliminary evidence of the antiviral activity of the vaccine. Although this analysis would clearly be exploratory, not incorporating the treatment interruption would be a lost opportunity, since patients will likely stop therapy on their own following completion of the vaccinations in a disorganized fashion that will not generate any useful data at all. Based on the safety data accumulated for DNA vaccines to date in both uninfected and HIV-1-infected individuals, the safety data available for VRC-HIVDNA009-00-VP in uninfected individuals, and the close monitoring proposed by this study design, a single antiretroviral treatment interruption following DNA vaccination should be safe for the participants in this study.

Individuals with treated acute HIV-1 infection have been demonstrated to have unusually high CD4+ T-cell responses but atypically low and narrowly focused CD8+ T cell responses. It is therefore of scientific importance to this study to follow the fractionated CD4+ and CD8+ T-cell responses, in addition to the total T-cell responses. Since tetramer assays and cellular fractionation techniques can be performedmost effectively on freshly isolated PBMC, Dr. Eric Rosenberg's and Dr. Dan Barouch's laboratories at the Harvard ACTU will perform the tetramer assays, CD4-depleted ELISPOT assays, and CD8-depleted ELISPOT assays for this study. Dr. Richard Koup’s laboratory at the VRC will perform the validated unfractionated ELISPOT and ICS assays for this study.

Testing for anti-dsDNA antibodies will be routinely performed. Generation of anti- dsDNA antibodies by plasmid DNA vaccines remains a theoretical concern, but this has not significantly occurred in DNA vaccine studies in humans to date.

# STUDY DESIGN

This study is a randomized, placebo-controlled, double-blinded trial to evaluate the safety and immunogenicity of the HIV-1 DNA vaccine VRC-HIVDNA009-00-VP in HIV-1-infected subjects on HAART. Healthy HIV-1-infected subjects who were treated with HAART during acute infection will be recruited and screened. Those determined to be eligible based on the inclusion and exclusion criteria described in section 4.0 will be enrolled in this 18-month study.

## 3.1 Study Overview

This study is divided into two parts. In the first part of the study, subjects will be randomized to receive either the vaccine or the placebo. In the second part of the study, subjects who did not reach a safety endpoint(as defined in section 9.2) will voluntarily undergo a supervised treatment interruption (STI).

*Part 1 – Therapeutic Vaccination*

For the first part of the study, subjects will receive 4 vaccinations with 4 mg DNA vaccine or placebo at weeks 0, 4, 8, and 24. Immunizations will be administered by the intramuscular route as one 1mL injection in the deltoid via needle-free Biojector® in the outpatient setting. The objectives are to assess the safety and immunogenicity of the DNA vaccine.

*Part 2 – Supervised Treatment Interruption*

All individuals completing the therapeutic vaccination phase (defined as completing at least three immunizations including the week 24 immunization) of the trial without reaching a safety endpoint will be given the opportunity to participate in the second part of the study and undergo a supervised discontinuation of HAART. The objective is to obtain preliminary exploratory data as towhether subjects receiving the DNA vaccine will have a lower setpoint viral RNA level as compared with subjects who receive a placebo. At week 30, subjects will discontinue all antiretroviral agents. Individuals taking non-nucleoside reverse transcriptase inhibitors (NNRTI) will be advised to switch to a protease inhibitor-containing regimen 1 week prior to the discontinuation of HAART to ensure that the washout period of antiretroviral agents is roughly equal. Alternatively, subjects will have the option instead to stop their NNRTI 24-72 hours before discontinuing HAART at the discretion of their primary physician.

## 3.2 Criteria to Restart HAART

Subjects will be monitored closely following the discontinuation of HAART. Criteria to restart HAART will be:

- A confirmed decline in CD4+ T lymphocyte counts from baseline(average of pre-entry and entry)of >50% or absolute CD4+ T lymphocyte counts of <250 cells/mm3 on two consecutive measurements.
- A confirmed HIV-1 RNA level of >100,000 copies/mL that is sustained at a level of >100,000 copies/mL for at least 8 weeks (+/- 5 days) following the first measurement exceeding 100,000 copies/mL.
- At the discretion of the study investigators if major abnormalities are noted in routine monitoring laboratories.
- If the subject experiences symptoms consistent with antiretroviral syndrome (see Appendix I) at the discretion of his or her primary physician.
- At the discretion of the subject and his or her primary physician at any time.

When individuals restart antiretroviral medications, an HIV-1 genotypic analysis will be performed. The same regimen prior to discontinuation of therapy will be administered unless a change is desired by the patient’s primary physician. The results of the HIV-1 genotypic analysis may be used to guide therapy changeswhen available.

The HIV-1 RNA levels averaged at weeks 48, 50, and 52 will be considered the new setpoint HIV-1 RNA level. At week 52, subjects will be restarted on HAART at the discretion of the subject’s primary physician according to current clinical guidelines (e.g., CD4+ < 350 cells/mm3 and HIV-1 RNA > 55,000 copies/mL) after notifying the team.

3.4 Criteria for Stopping the Study

Details are described in section 9.4.

# SELECTION AND ENROLLMENT OF SUBJECTS

## 4.1 Inclusion Criteria

4.1.1 Subjects with treated acute HIV-1 infection defined as initiating HAART during the acute retroviral syndrome and who were diagnosed by a positive HIV-1viral load and a negative or indeterminate Western blot.

4.1.2 Prior antiretroviral therapy defined as a minimum of 6 months of treatment with HAART. (HAART is defined as a combination of at least 2 antiretroviral agents.)

4.1.3 CD4+ cell count >350 cells/mm3 for a period of 6 months prior to study entry at least at 3 different timepoints (one of which must be obtained at screening) obtained at any laboratory that possesses a CLIA certification or its equivalent.

4.1.4 Screening CD4+ cell count >350 cells/mm3 within 30 days prior to study entry obtained at any laboratory that possesses a CLIA certification or its equivalent.

4.1.5 HIV-1 RNA levels <50 copies/mLfor a period of 6 monthsprior to study entry at least at 3 different timepoints (one of which must be obtained at screening) by any laboratory that possesses a CLIA certification or its equivalent.

4.1.6 Screening HIV-1 RNA levels <50 copies/mL within 30 days prior to study entry obtained at any laboratory that possesses a CLIA certification or its equivalent.

4.1.7 Laboratory values obtained within 30 days prior to study entry:

- Absolute neutrophil count (ANC) ≥ 750/mm3.
- Hemoglobin ≥ 7.0 g/dL.
- Platelet count ≥ 50,000/mm3.
- Creatinine ≤ 3 x ULN.
- AST (SGOT), ALT (SGPT), and alkaline phosphatase ≤ 5 x ULN.
- Total bilirubin ≤ 2.5 x ULN.
- Serum lipase≤ 1.5 x ULN.

4.1.8 Female study volunteers of reproductive potential (defined as girls who have reached menarche or women who have not been post-menopausal for at least 24 consecutive months, i.e. who have had menses within the preceding 24 months, or have not undergone surgical sterilization (e.g. hysterectomy, bilateral oophorectomy, or salpingotomy) must have a negative serum or urine pregnancy test performed within 3 days before initiating study-specified medication.

If participating in sexual activity that could lead to pregnancy, the female study volunteer must use a form of contraception listed below while receiving protocol-specified medication and for 6 months after stopping the medication. At least one of the following methods MUST be used appropriately (with or without hormone-based method):

- Condoms1 (male or female) with or without a spermicidal agent
- Diaphragm or cervical cap with spermicide
- IUD

1 Condoms are recommended because their appropriate use is the only contraception method effective for preventing HIV transmission.

If the female volunteer is not of reproductive potential (girls who have not reached menarche, women who have been post-menopausal for at least 24 consecutive months, or women who have undergone surgical sterilization, (e.g. hysterectomy, bilateral oophorectomy, or salpingotomy) she is eligible without requiring the use of contraception. Patient-reported history is acceptable as documentation of sterilization, contraceptive methods, or menopause.

4.1.9 Men and women aged 18-50 years*.*

4.1.10 Ability and willingness of subject or legal guardian/representative to give written informed consent.

## 4.2 Exclusion Criteria

4.2.1 History of serious adverse reactions to vaccines including anaphylaxis and related symptoms such as hives, respiratory difficulty, angioedema, and/or abdominal pain**,** except for subjects who have a history of an adverse reaction to pertussis vaccine as a child may enroll.

- - 1. History of CD4+ counts <250 cells/mm3, opportunistic infections, or AIDS-defining illnesses.

- - 1. History of autoimmune disease, immunodeficiency (other than HIV infection), asthma, diabetes(including Type II diabetes or insulin resistance)requiring insulin or oral hypoglycemics, thyroid disease, bleeding disorder, active malignancy, viral hepatitis, or asplenia.
    2. HBV sAg, HCV Ab, or RPR positive.
    3. Suspected allergy or adverse reaction to any components of the study agent.
    4. Changes in antiretroviral regimen within 6 months prior to entry due to virologic failure (not including toxicities).
    5. Previous participation in supervised treatment interruptions (STIs).
    6. Pregnancy or breast-feeding.
    7. Receipt of live attenuated vaccines or investigational research agents within 30 days prior to study entry.
    8. Receipt of blood products within 120 days prior to study entry.
    9. Receipt of immunoglobulin within 60 days prior to study entry.
    10. Receipt of medically indicated subunit or killed vaccines (e.g., influenza, pneumococcal, or allergy treatment with antigen injections) within 14 days prior to study entry.
    11. Receipt of prior experimental HIV vaccines. Individuals that received a placebo in a prior HIV vaccine trial are not excluded, provided that they did not receive a sham vector or an adjuvant.

- - 1. Receipt of immunosuppressive medications within the past 6 months (e.g., oral/parenteral/inhaled corticosteroids, and/or cytotoxic medications). NOTE: The following will be allowed: corticosteroid nasal spray for allergic rhinitis; topical corticosteroids for acute, uncomplicated dermatitis; over the counter medications for acute, uncomplicated dermatitisfor a period not longer than 14 days.
    2. Current anti-TB prophylaxis or therapy.
    3. Active drug or alcohol use or dependence that, in the opinion of the investigator, would interfere with adherence to study requirements.
    4. Serious illness (requiring systemic treatment and/or hospitalization) until subject either completes therapy or is clinically stable on therapy, in the opinion of the investigator, for at least 14 days prior to study entry.
    5. Anti-dsDNA antibodygreater than the upper limit of normal for the testing laboratory.

## 4.3 Study Enrollment Procedures

- - 1. Prior to implementation of this protocol, sites must have the protocol and consent form approved by their local institutional review board (IRB). Sites must be registered with and approved by the DAIDS**/**Regulatory Compliance Center Protocol Registration Office. Site registration must occur before any subjects can be enrolled in this study.

       Once a candidate for study entry has been identified, details will be carefully discussed with the subject. The subject will be asked to read and sign the consent form that was approved by both the AACTU’s IRB and the DAIDS/Regulatory Compliance Center Protocol Registration Office.
    2. The Division of AIDS has concluded that this protocol does NOT meet Federal requirements governing prisoner participation in clinical trials and should NOT be considered by local IRBs for the recruitment of prisoners.

4.3.3 All subjects will be randomized at entry using standard procedures by the AACTG Data Management Center.

# STUDY TREATMENT

Study treatment is defined as the vaccine, VRC-HIVDNA009-00-VP.

## 5.1 Regimen, Administration, and Duration

5.1.1 Regimen

Part 1: Therapeutic Vaccination (weeks 0 to 30)

Subjects will remain on HAART (defined as at least 2 antiretrovirals) for the first 30 weeks of the study. At entry, subjects will be randomized to receive one of the following:

- Arm A: VRC-HIVDNA009-00-VP 4mg/1 mL IM at weeks 0, 4, 8, and 24.
- Arm B: Phosphate Buffered Saline (PBS) 1mL IM at weeks 0, 4, 8, and 24.

Part 2: Supervised Treatment Interruption

In the second part of the study, at study week 30, subjects who did not reach a safety endpoint will undergo a supervised treatment interruption. Subjects will be monitored closely. If subjects reach the criteria defined in section 3.2 they will restart antiretroviral therapy. At study week 52, subjects will restart HAART at the discretion of their primary care physician as defined in section 3.2.

5.1.2 Administration

The Biojector® 2000 Needle-Free Injection Management System will be used as directed by the manufacturer. Neither the material being injected nor injection site skin preparation require deviation from standard procedures. The injection site is disinfected and allowed to dry completely. The skin around the injection site is held firmly while the syringe is placed against the injection site at a 90° angle. The actuator is pressed and the material is released into the muscle. After the injection, the site is covered with a sterile covering and pressure applied. The Biojector® device utilizes sterile, single-use syringes for variable dose, up to 1 mL, medication administration. The study agent is delivered under pressure by a compressed carbon dioxide (CO2) gas cartridge that is stored inside the Biojector®. When the Biojector® actuator is depressed, CO2 is released, causing the plunger to push the study agent out of the sterile syringe through the skin and into the underlying tissue. The study agent is expelled through a micro- orifice at high velocity in a fraction of a second to pierce the skin. The CO2 does not come in contact with the injectate, and the syringe design prevents any back splatter or contamination of the device by tissue from the subject.

A 1 mL injection will be administered intramuscularly into the deltoid muscle at weeks 0, 4, 8, and 24.

5.1.3 Duration

The total duration of the study is 72 weeks for each subject.

## 5.2 Product Formulation and Preparation

5.2.1 Product Formulation

The investigational vaccine, VRC-HIVDNA009-00-VP, is formulated in Phosphate Buffered Saline (PBS) at a pH of 7.2. It is supplied in 2 mL glass vials containing 1.3 mL of a clear, colorless, isotonic, sterile solution at a concentration of 4 mg/mL. The product must be stored frozen (-10° to -70° C). Vials should not be refrozen after thawing. Vials are intended for single use only.

Placebo Control (Phosphate Buffered Saline, PBS) is supplied in 2 mL glass vials containing 1.3 mL of a clear, colorless, isotonic sterile solution. The product must be stored at controlled room temperature, 20° to 25°C (68° to 77°F).

5.2.2 Product Preparation

Using a Biojector® syringe, withdraw 1 mL of vaccine or placebo from the vial. The Biojector® 2000 Needle Free injection System will be used as directed by the manufacturer.

## 5.3 Product Supply, Distribution, and Pharmacy

5.3.1 Study Product Acquisition

VRC-HIVDNA009-00-VP is manufactured by Vical Incorporated (SanDiego, CA). Phosphate Buffered Saline (PBS) is manufactured by Bell- More Labs, Incorporated. Both will be provided by the Vaccine Research Center (VRC), National Institute of Allergy and Infectious Diseases, NIH. The Biojector® will be supplied by Bioject, Inc. The AACTG pharmacist can obtain the study agents and Biojector® syringes from the Clinical Research Products Management Center (CRPMC) by following the procedures in the manual, Pharmacy Guidelines and Instructions for DAIDS Clinical Trials Networks, in the section Study Product Control.

5.3.2 Study Product Accountability

The AACTU pharmacist is required to maintain complete records of all study products received from the NIAID Clinical Research Products Management Center and subsequently dispensed. All unused study products must be returned to the NIAID Clinical Research Products Management Center after the study is completed or terminated. The procedures to be followed are provided in the manual, Pharmacy Guidelines and Instructions for DAIDS Clinical Trials Networks, in the section Study Product Control.

5.3.3 Study Product Disposal

Any empty vials, unused portions of vials, and expired Biojector® syringes should be discarded in a biohazard containment bag and incinerated.

## 5.4 Concomitant Medications

- - 1. Required Medications

Subjects are required to maintain a fully suppressive HAARTregimen (defined as 2 or more antiretroviral medications) throughout the vaccination period, until the supervised treatment interruption. These medications will not be provided by the study.

- - 1. Prohibited Medications

Other vaccines of any type, investigational research agents, immunoglobulin, antineoplastic agents, glucocorticoids (other than corticosteroid nasal spray for allergic rhinitis; topical corticosteroids for acute, uncomplicated dermatitis; over the counter medications for acute, uncomplicated dermatitis for a treatment period not longer than 14 days), or other immunomodulator medications (other than NSAIDS which will be allowed for any length of time).

# CLINICAL AND LABORATORY EVALUATIONS

## 6.1 Schedule of Events: Part 1

| Evaluation | Screening | Pre-Entry | Entry | Week 1: 1-2 days post vaccine | Week 2 | Week 4 | Week 4:1-2 days post vaccine | Week 6 | Week 8 | Week 8:1-2 days post vaccine | Week 10 | Week16 | Week 24 | Week 24:1-2 days post vaccine | Week 26 |
| --- | --- | --- | --- | --- | --- | --- | --- | --- | --- | --- | --- | --- | --- | --- | --- |
| Documentation of HIV | X |  |  |  |  |  |  |  |  |  |  |  |  |  |  |
| Medical/Medication History | X |  |  |  |  |  |  |  |  |  |  |  |  |  |  |
| Concomitant Medications |  |  | X |  | X | X |  | X | X |  | X | X | X |  | X |
| Nadir CD4+ | X |  |  |  |  |  |  |  |  |  |  |  |  |  |  |
| Complete Physical Exam | X |  |  |  |  |  |  |  |  |  |  |  |  |  |  |
| Targeted Physical Exam |  |  | X |  | X | X |  | X | X |  | X | X | X |  | X |
| Hematology | X | X | X |  | X | X |  | X | X |  | X | X | X |  | X |
| Chemistry | X |  | X |  | X |  |  | X |  |  | X | X |  |  | X |
| Urinalysis | X |  | X |  | X | X |  | X | X |  | X | X | X |  | X |
| Pregnancy Testing**1** | X | X | X |  |  | X |  |  | X |  |  |  | X |  |  |
| CD4/CD8 | X | X | X |  | X | X |  | X | X |  | X | X | X |  | X |
| Cellular & Humoral Responses2 |  | X | X |  |  |  |  | X |  |  | X |  | X |  | X |
| Real Time Immunology Assays2 |  | X | X |  |  |  |  | X |  |  | X |  | X |  | X |
| HIV-1 RNA (real time) | X | X | X |  | X | X |  | X | X |  | X | X | X |  | X |
| HBV sAg, HCV Ab, RPR, HLA typing | X |  |  |  |  |  |  |  |  |  |  |  |  |  |  |
| Vaccine Administration |  |  | X |  |  | X |  |  | X |  |  |  | X |  |  |
| Telephone Contact |  |  |  | X |  |  | X |  |  | X |  |  |  | X |  |

**1**To be performed at any time pregnancy is suspected.

2Refer to Appendix II for the amount of blood required at a particular visit. NOTE: These will not be performed if the subject becomes pregnant.

6.1 Schedule of Events: Part 2

| Evaluation | | 30 | | | 32 | 34 | 36 | 38 | 40 | 42 | 44 | 46 | 48 | 50 | 52 | 56 | 60 | 64 | 68 | 72 | | Premature Disc. |
| --- | --- | --- | --- | --- | --- | --- | --- | --- | --- | --- | --- | --- | --- | --- | --- | --- | --- | --- | --- | --- | --- | --- |
| HAART Discontinuation | | X | | |  |  |  |  |  |  |  |  |  |  |  |  |  |  |  |  | |  |
| Targeted Physical Exam | | X | | | X | X | X | X | X | X | X | X | X | X | X | X | X | X | X | X | | X |
| Concomitant Medications | | X | | | X | X | X | X | X | X | X | X | X | X | X | X | X | X | X | X | | X |
| Hematology | | X | | | X | X | X | X | X | X | X | X | X | X | X | X | X | X | X | X | | X |
| Chemistry | |  | | | X |  | X |  | X |  | X |  | X |  | X |  | X |  | X |  | | X |
| Urinalysis | | X | | | X | X | X | X | X | X | X | X | X | X | X | X | X | X | X | X | | X |
| Pregnancy Testing1 | | X | | | X |  | X |  | X |  | X |  | X |  | X | X | X | X | X | X | | X |
| CD4/CD8 | | X | | | X | X | X | X | X | X | X | X | X | X | X | X | X | X | X | X | | X |
| Cellular & Humoral Responses2 | |  | | |  | X |  |  |  | X |  |  |  | X |  |  | X |  |  | X | | X |
| Real Time Immunology Assays2 | |  | | |  | X |  |  |  | X |  |  |  | X |  |  | X |  |  | X | | X |
| HIV-1 RNA (real time) | | X | | | X | X | X | X | X | X | X | X | X | X | X | X | X | X | X | X | | X |
| HIV-1 Genotypic Analysis | | | Prior to re-starting HAART | | | | | | | | | | | | | | | | | | | |
| Restart HAART |  | | | See section 3.2 | | | | | | | | | | | | | | | | |  | |

1To be performed at any time pregnancy is suspected.

2Refer to Appendix II for the amount of blood required at a particular visit.NOTE: These will not be performed if the subject becomes pregnant.

## 6.2 Definitions for Schedule of Events – Timing of Evaluations

6.2.1 Pre-randomization Evaluations

Occur prior to the subject taking any study medications, treatments, or interventions.

Screening

Screening evaluations to determine eligibility must be completed within 30 days prior to study entry unless otherwise specified*.*

Pre-Entry

Pre-entry evaluations must be completed within 14 days prior to entry evaluations and at least 24 hours after screening.

6.2.2 On-Study Evaluations

Evaluationsoccur after randomization. Study visits must be scheduled on the weeks indicated in the Schedule of Events ± 7days.

Entry

Entry evaluations must occur at least 24 hoursafter pre-entry evaluation. Subject must begin treatment within 72 hours of randomization.

6.2.3 Evaluations for randomized subjects who do not start study treatment

Subjects who do not start study treatment will be taken off study with no further evaluations required. However, all case report forms (CRFs) up to and including entry (week 0) must be completed and keyed into the database.

6.2.4 Study Completion Evaluations

The week 72 evaluations will be completed at the subject’s final on study visit.

6.2.5 Pregnancy

Subjects who become pregnant and have not completed all vaccines will NOT receive any further vaccinesor participate in the treatment interruption part of the study. However, the subject should remain on study and receive safety follow-upfor the remainder of the study visits. The evaluations will include hematology, chemistry, CD4+ cell counts, and viral load in addition to a targeted physical exam.

## 6.3 Special Instructions and Definitions of Evaluations

6.3.1 Documentation of Acute HIV-1 Infection

Acute HIV-1 infection, as documented by a positive HIV-1 viral load, and a negative or indeterminate Western blotat any time prior to screening.

6.3.2 Medical History

A medical history must be present in source documents. Record all diagnoses identified by the AACTG criteria for clinical events and other diseases on the CRF.  For the current criteria, refer to the appendix identified in the study CRF. Any allergies to any medications and their formulations must be documented.

6.3.3 Medication History

A medication history must be recorded on the CRF, including:

- Complete HIV treatment history, including start and stop dates of any antiretroviral medication (estimated if the exact dates cannot be obtained), immune-based therapy, or HIV-related vaccines, including blinded study medications.
- Complete treatment history of any prescription medications taken for the treatment or prophylaxis of opportunistic infections, including actual or estimated start and stop dates.
- All prescription medications (in addition to those noted above) taken within 30 days prior to entry, including actual or estimated start and stop dates.
- Nonprescription medications, taken within 30 days prior to entry*.* Include actual or estimated start and stop dates.
- Alternative therapies taken within 30 days prior toentry. Include actual or estimated start and stop dates.

6.3.4 Concomitant Medications

All concomitant medications taken since the last report will be recorded in the source documentation. The source document must include start dates, stop dates, and dose changes.

Please note that only antiretroviral therapyand prescription medicationswill be recorded on the CRFsincluding start dates, stop dates, dose changes, and current status.

6.3.5 Study Treatment Modifications

All modifications to study vaccine including initial doses, patient-initiatedand/or protocol-mandated interruptions, modifications, and permanent discontinuation of treatment will be recorded on the CRFs.

6.3.6 Nadir CD4+

The subject’s prior nadir CD4+ cell count (absolute value and date) should be documented when possible with a copy of the nadir CD4+ cell count report and recorded on the CRF. If this documentation is not available, then subject recollection will suffice. For subjects who do not know the exact nadir value and for whom there is no source documentation, then recall of the categorical nadir (e.g., <200, <250, <300 cells/mm3) will suffice.

6.3.7 Clinical Assessments

Complete Physical Exam

Complete physical examination is required at screening and includes examination of the skin, head, mouth, and neck; auscultation of the chest; cardiac exam; and abdominal exam. The complete physical exam will also include signs and symptoms, diagnoses, and vital signs(temperature, blood pressure, pulse).

Targeted Physical Exam

A targeted physical examination, to be driven by any signs or symptoms previously identifiedor new that the subject has experienced since the last visit.

This exam should also include weight, vital signs, signs and symptoms, and diagnoses at all visits.

Weight

Recorded at all visits on the CRFs.

Height

Recorded only at entry.

Signs and Symptoms

At entry, record all signs and symptoms occurring within 30 days prior to entry on the CRFs. After entry, all signs and symptoms, regardless of grade, must be recorded on the CRFs within 48 hours.

Refer to the Division of AIDS Table for Grading Adult Adverse Experienceswhich can be found on the Regulatory Compliance Center (RCC) Web site: [http://rcc.tech-res-intl.com](http://rcc.tech-res-intl.com/).

Diagnoses
All confirmed and probable diagnoses made since the last visit will be recorded in the CRF according to the current AACTG criteria.For the current criteria, refer to the appendix identified in the study CRF. Record deaths on the appropriate CRF and key within 48 hours.

Vital Signs

Temperature, pulse, and blood pressure collectedat all visitson the CRF*.*

6.3.8 Laboratory Evaluations

All laboratory values, regardless of grade, are to be recorded on the CRFs. After entry, all laboratory results must be recorded on the CRFs within 48 hours throughout the course of the study.

Refer to the Division of AIDS Table for Grading Adult Adverse Experienceswhich can be found on the Regulatory Compliance Center (RCC) Web site: [http://rcc.tech-res-intl.com](http://rcc.tech-res-intl.com/).

Hematology:

Hemoglobin, mean corpuscular volume (MCV), white blood cell count (WBC), differential WBC, absolute neutrophil count(ANC), platelets.

Blood Chemistries

Lipase, glucose, creatine kinase, sodium, potassium, BUN, creatinine, ALT (SGPT), AST (SGOT), total bilirubin, anti-dsDNA.

Urinalysis

Urine dipstick will be performed. If abnormalities are found, a specimen for complete urinalysis must be done at the local laboratory. Results will be recorded on the CRF.

Pregnancy Test

For women with reproductive potential: Serum or urine β-HCG (urine test must have a sensitivity of 25-50 mIU/mL).Results will be recorded on the CRF.

6.3.9 Immunologic Studies

CD4+/CD8+

Obtain absolute CD4+/CD8+ cell count and percentages within 30 daysprior toentry from a certified laboratory. All laboratories must possess a CLIA certification or its equivalent in order to perform CD4+ cell count testing that is used for patient management. For pre-entry and on study CD4+/CD8+ cell count,the laboratory also must be certified for protocol testing by the DAIDS Immunology Quality Assurance (IQA) Program.

Evaluations for CD4+ and CD8+ counts and subset percentage evaluations should be performed at the same AACTG-certified laboratory, if possible, for baseline calculation and throughout the course of the study.

Because of the diurnal variation in CD4+/CD8+ cell count, determinations for individual subjects should be obtained consistently in either the morning or the afternoon throughout the study, if possible.

Note: Each time a CD4+/CD8+ measurement is obtained, the local laboratory must perform a WBC and differential from a sample obtainedat the same time.

6.3.10 Immunology Assays (See Appendix II)

Cellular and humoral responses

The following validated immunologic studies will be performed at the VRC:

- Cellular immune responses by pooled peptide IFN-γ ELISPOT and ICS assays specific for Gag, Pol, Nef, and Env using unfractionated PBMC
- Humoral immune responses by ELISA and neutralizing antibody assays

Real time assays

In addition, the following ancillary immunologic studies will be performed to study the immune responses in greater detail for research purposes at the Harvard ACTUusing freshly isolated and fractionated PBMC:

- Fractionated CD8+ T-lymphocyte responses by ELISPOT assays using CD4+-depleted PBMC and tetramer assays in HLA-appropriate subjects
- Fractionated CD4+ T -lymphocyte responses by ELISPOT assays using CD8+-depleted PBMC, lymphoproliferation assays (LPA), and tetramer assays in HLA-appropriate subjects
- Phenotyping of central and effector memory CTL by flow cytometry

6.3.11 Virologic Studies

Plasma HIV-1 RNA

Screening HIV-1 RNA testsmust be performed using the Roche Ultrasensitive method by a laboratory that possesses CLIA certification or its equivalent. Eligibility will be determined based on the screening value. The baseline value will be the mean of the pre-entry and entry determinations, which must have been obtained at least 24 hoursapart. On-study HIV-1 RNA specimens must be sent to the designated laboratory (see Appendix II).

HIV-1 genotypic analysis

To be done prior to restarting HAART at the Harvard AACTU virology support laboratory (VSL).

In addition, viral sequencing to assess for viral evolution may also be done on frozen samples at the Harvard AACTU VSL.

6.3.12 Other Laboratory Studies

HBV sAg, HCV Ab, and RPR to be done at the local clinical lab and documented in the source documents. HLA typing (class 1) to be done at the local clinical laboratory and results recorded on the CRFs.

6.3.13 Vaccine Administration

VRC-HIVDNA009-00-VP vaccine or placebo will be administered as intramuscular injections on the appropriate inoculation days. Each subject will receive one injection in the deltoid for each vaccination. Intramuscular injections will be performed using the needle-free Biojector® device according to the manufacturer’s directions.

Following each immunization, subjects will be observed for 60 minutes in the clinic. Temperatures, vital signs, and reactions will be assessed at 15, 30, and 60 minutes post-immunization. If erythema, edema, or induration are present, diameters will be measured at each time point. All subjects with Grade > 3 local or systemic reaction at the 60 minute post-immunization timepoint will return to the clinic the day after immunization, or sooner if clinically indicated for evaluation.

Subjects will take and record their temperatures the same evening and for 2 days following immunization, and record any symptoms they experience.

6.3.14 Telephone Contact

A telephone contact will be made 1-2 days after each vaccination to assess vaccine tolerability. Subjects will return for additional follow-up visits if clinically indicated.

6.3.15 HAART Discontinuation

At week 30, subjects will discontinue all antiretroviral agentsif they have not met a safety endpoint. Individuals taking non-nucleoside reverse transcriptase inhibitors (NNRTI) will be advised to switch to a protease inhibitor- containing regimen 1 week prior to the discontinuation of HAART to ensure that the washout period of antiretroviral agents is roughly equal. Alternatively, subjects will have the option instead to stop their NNRTI 24-72 hours before discontinuing HAART at the discretion of their primary physician. Subjects will be monitored closely following the discontinuation of HAART. When individuals restart antiretroviral medications, an HIV-1 genotypic analysis will be performed. The same regimen prior todiscontinuation of therapy will be administered unless a different regimen is indicated based on genotypic analysis (if the results are available) or desired by the subject’s primary physician.

Subjects who become pregnant prior to week 30 will not undergo STI. If subjects become pregnant while in part 2 of the study, they may continue on-study after consultation with their primary care physician and will be followed for safety evaluations including hematology, chemistry, CD4+ cell counts, and viral load in addition to a targeted physical exam.

## 6.4 Off-Vaccine Requirements

Participants who are discontinued from additional study injections will continue to be followed according to the schedule to further evaluate safety and monitor adverse events. The evaluations will include hematology, chemistry, CD4 cell counts, and viral load in addition to a targeted physical exam. Subjects will only participate in the supervised HAART discontinuation if they received at least three immunizations including the week 24 immunization and have not reached a safety endpoint.

Additional safety monitoring and reporting of serious adverse experiences (SAEs) continues to be required upon completion or discontinuation of study treatment regardless of whether a protocol follow-up period is scheduled to occur. As specified in the current Division of AIDS *Serious Adverse Experience (SAE) Reporting Manual,* adverse experiences occurring during the immediate 6 month period after the last dose of study treatment which meet SAE reporting requirements must be reported to the DAIDS Regulatory Compliance Center SAE Office. Additionally, after 6 months OFF study treatment, there are four types of events that must be reported to the SAE Office if the relationship to the study drug is assessed by the site physician as definitely, possibly, or unable to judge: DEATHS, NEW ONSET CANCERS, CONGENITAL ANOMALIES, AND PERMANENT DISABILITIES.

# TOXICITY MANAGEMENT

For documenting reactions to study vaccines, use the following table:

TABLE 1: Local reaction assessments

| Grade | Pain and/or Tenderness Grading Scale | Serious Adverse Event Reporting System Cutaneous |
| --- | --- | --- |
| 0 = None | No pain or tenderness | N/A |
| 1 = Mild | Minimal pain or tenderness, no limitation of arm use | Erythema, indurationor edema less than 15 cm x 15 cm (225 cm sq) |
| 2 = Moderate | Notable pain or tenderness, some limitation of use of arm | Erythema, induration or edema ≥ 15 x 15 cm  (225 cm sq) |
| 3= Severe | Extreme pain or tenderness, complete limitation of use of arm | Ulceration or superinfection or phlebitis |
| 4= Potentially Life-threatening | N/A | Necrosis of the skin |

- Local reactions

Local reactions will be graded using the scales in Table 1. Discomfort will be graded according to the Pain and/or Tenderness Grading Scale. Cutaneous reactions will be graded according to the Serious Adverse Event Reporting System (cutaneous).

- Local reactions Grade 1 or 2 on the scales

Local reactions of mild (Grade 1) or moderate (Grade 2) severity will usually resolve spontaneously. If needed, they may be managed with local application of cold packs, oral acetaminophen, oral nonsteroidal anti-inflammatory agents, or a combination of these measures as appropriate.

- Local reactions Grade 3 or 4 on the scales

The protocol team should be notified of all Grade 3 or 4 local toxicities within 48 hours of occurrencefor consideration of modification or discontinuation of immunizations. For example, if a subject develops an intolerable local swelling (Grade 3 on the pain/tenderness scale) during the 72 hours after immunization, the protocol team should be contacted.

For Grade 4 local reactions, definitive medical and/or surgical intervention should be undertaken as appropriate.

- Systemic reactions

Systemic reactions will be graded according to the Division of AIDS Table for Grading Adult Adverse Experiences which can be found on the Regulatory Compliance Center (RCC) Web site: [http://rcc.tech-res-intl.com](http://rcc.tech-res-intl.com/).

The protocol team should be contacted within 48 hours for any non-local Grade 3 or 4 reactions (for example, elevated temperatures following immunizations) thought definitely, possibly, or probably related to vaccination. Criteria for discontinuation of additional vaccinations are described in section 8.0.

# CRITERIA FOR DISCONTINUATIONOF VACCINE

- Grade 3 or higherlocal or systemic event classified as “possibly,” “probably,” or “definitely” associated with immunization.
- 2 consecutive viral loads ≥ 400 copies/mL while on HAART.
- 2 consecutive absolute CD4+ counts ≤ 250 cells/mm3 while on HAART.
- 2 consecutive CD4+ counts more than 50% below the CD4+ count at baseline (average of pre-entry and entry) while on HAART.
- Development of a serious intercurrent illness that is not expected to resolve prior to the next scheduled immunization.
- Requirement for prohibited concomitant medications (see section 5.4).
- Repeated failure to comply with protocol requirements.
- Missing 2 doses of study vaccine.
- Pregnancy or breastfeeding.
- Request by the subject to withdraw.
- Request of the primary care provider if s/he thinks the study is no longer in the best interest of the subject.
- Clinical reasons believed life threatening by the physician, even if not addressed in the toxicity management section of the protocol.
- Subject judged by the investigator to be at significant risk of failing to comply with the provisions of the protocol as to cause harm to self or seriously interfere with the validity of the study results.
- At the discretion of the AACTG, FDA, NIAID,the VRC, or investigator.

# STATISTICAL CONSIDERATIONS

## General Design Issues

This study is a phase I**/**II, randomized, placebo-controlled, double-blind trial to evaluate the safety of the HIV-1 DNA vaccine VRC-HIVDNA009-00-VP.  The study will enroll 20 healthy HIV-1-infected subjects who were treated with antiretroviral therapy during acute HIV infection. Subjects will be randomized in a 1:1 fashion to vaccine versus placebousing permuted blocks.  The primary objective is to evaluate the safety of the vaccine. Immunogenicity and viral load setpoint during antiretroviral treatment withdrawal will be assessed in exploratory analyses. The vaccine dose used in this study has been evaluated in an NIH-sponsored dose-escalation study of this vaccine in HIV-negative subjects (VRC-004).  Randomized subjects who never receive an injection will be replaced and not analyzed. Accrual is anticipated to take approximately six months.

Initial analyses of the immunogenicity data, by arm and with blinded subject identifiers, is planned to occur after all subjects have completed their week 26 evaluations. Subjects will be unblinded approximately 3 months after the last subject’s week 26 evaluations.

## 9.2 Endpoints

- - 1. Primary SafetyEndpoint

The primary endpoint is the development of:

- a Grade 3 or 4 sign/symptom or laboratory abnormality,or death, that is at least possibly related to vaccine, based on team assessment (blinded to the vaccine/placebo status of the subject), or
- 2 consecutive viral loads ≥ 400 copies/mL while receiving HAART, or
- 2 consecutive absolute CD4+ counts ≤ 250 cells/mm3 while receiving HAART, or
- 2 consecutive CD4+ counts more than 50% below the CD4+ count at baseline (average of pre-entry and entry).

Only events that occur on or subsequent to the first vaccine and within 24 weeks of the last vaccine administration will be considered.For analysis purposes,laboratory abnormalities which do not have a DAIDS toxicity grading scale will not contribute to the primary safety endpoint.

- - 1. Secondary Endpoints
- Tolerability.  Receipt of the full schedule of four vaccines.
- Viral load setpoint.  The viral load setpoint is defined as the average of the log10 viral load measures at weeks 18, 20, and 22 after antiretroviral treatment (HAART) withdrawal (study weeks 48, 50, and 52).  If a subject reinitiates HAART before this time or otherwise does not have an off-HAART viral load 18-22 weeks after withdrawing HAART, the viral load setpoint will be defined as the average of the last two off-HAART log10 viral loads that are at least 12 weeks after withdrawing HAART. Otherwise, the viral load setpoint will not be defined (for example, if a subject reinitiates HAART before 12 weeks off-treatment).
- ELISPOT. A positive vaccine-elicited ELISPOT response for an antigen will be defined as a 2-fold increase from baseline that is also ≥ 100 spots/106 PBMCs. A total ELISPOT response variable will be determined for each subject as the number of clade-B antigens (gag, pol, nef, env) for which a positive vaccine-elicited ELISPOT response was observed on atleast 2 post-baselinetimepoints.
- Intracellular cytokine staining (ICS).  A positive vaccine-elicited ICS response for an antigen will be defined as a 2-fold increase from baseline that is also ≥ 300 spots/106 PBMCs. A total ICS response variable will be determined for each subject as the number of clade-B antigens (gag, pol, nef, env) for which a positive vaccine-elicited ICS response was observed on at least 2 post-baseline timepoints.
  - 1. Ancillary assays
- LPA.  The LPA data will be described in terms of the stimulation
  index (SI), which is defined as the median counts per minute (CPM)
  in the stimulated replicates divided by the median CPMs in the
  appropriate control replicates.  If an observed SI is less than one,
  a value of one will be imputed for the analyses.  An LPA response is
  defined as an SI>3 which is also at least a 3-fold increase from baseline.
- Tetramer
- Neutralizing antibody

## 9.3 Sample Size and Accrual

With 10 active vaccine recipients in the primary safety analysis, the probability of detecting at least one safety endpoint is 80% if the true rate is 15%.  The probability of detecting at least one safety endpoint is only 34% if the true rate is 4%. Table 2shows the probability of detecting at least one safety endpoint, based on 10 subjects, for varying true underlying rates.

Table 2

| True subject-specific probability of a safety endpoint | Probability of detecting at least one safety endpoint based on 10 subjects |
| --- | --- |
| 0.02 | 0.18 |
| 0.04 | 0.34 |
| 0.06 | 0.46 |
| 0.08 | 0.57 |
| 0.10 | 0.65 |
| 0.15 | 0.80 |
| 0.20 | 0.89 |

The secondary endpoints of immunogenicity are exploratory in nature. For the ELISPOT (or ICS) endpoint, treatment-arm-specific probabilities of observing 0, 1, 2, 3, or 4 for the total response are described, which yield 80% power. For example, if the probabilities are 0.36, 0.3, 0.2, 0.1, and 0.04 in the control arm (that is, higher probability for lower total response) and 0.04, 0.1, 0.2, 0.3, and 0.36 in the active vaccine arm (that is, higher probability for higher total response), there is 80% power to detect a treatment arm difference, based on an alpha=0.05 2-sided Wilcoxon ranksum test with n=10 evaluable subjects per arm. Power was determined by numerical simulation with 10,000 replications.

Power may be limited for detecting significant differences in the viral load setpoint. For example, with nine evaluable subjects in each arm (assuming that the viral setpoint is unavailable in 2 subjects and that the unavailability is random and unrelated to treatment arm), the study has 80% power to detect a 1.5 standard deviation treatment arm difference in log10 viral setpoint, based on an alpha=0.05 2-sided Wilcoxon ranksum test. Power was determined by numerical simulation of normally distributed viral setpoints, with 10,000 replications. To translate this effect into log10 HIV-1 RNA copies/mL requires an estimate of the between-subject standard deviation of the viral setpoint. There is a range of standard deviations reported in the literature, from SD=0.52 (8 subjects, at end of third STI, [30]), SD=0.60 (128 subjects, either not receiving or on stable antiretroviral therapy, [31]), to SD=0.84 (9 subjects, at end of third STI, [32]). For example, assuming the standard deviation to be 0.60 in each arm (based on the estimate from the study with the most subjects), this study would have 80% power to detect a (two-sided) shift in the viral setpoint of 0.9 log10 copies/mL between the two arms. Greater variability in viral setpoints would imply lower power to detect a 0.9 log10 treatment effect.

## 9.4 Monitoring

Safety monitoring of adverse events will occur every 2 weeks. This will include team review of adverse events (including all reported signs/symptoms and laboratory abnormalities) and team assessment as to the possible relationship of adverse events to the study vaccine. The adverse event report will not indicate the randomized arm of the subjects. Monitoring will also include early treatment discontinuations, early study discontinuations and a summary of any subjects who reach week 30 but do not withdraw antiretroviral treatment. In addition, subjects’ baseline characteristics will be reviewed periodically during accrual.

An Immunology-Research Agenda Committee appointed Study Monitoring Committee will be convened to review the study data, broken down by vaccine/placebo arm, if 4 or more subjects (or 2 of the first 4 subjects) are observed to have Grade 3 events, that are at least possibly related to vaccine (based on team assessment), or if 1 subject is observed to have a Grade 4 event that is at least possibly related to vaccine (based on team assessment). Accrual will be halted if such a Grade 4 event or death is observed. A Study Monitoring Committee may also be convened if a reason is identified by the Medical Officer or study statistician in consultation with the team. Based on a review of the safety data, by treatment arm, the Study Monitoring Committee will determine whether to stop or modify the study. Also, the Study Monitoring Committee will review the study approximately 12 months after the first subject receives injections, and yearly thereafter while the study is on-going.

## 9.5 Analyses

9.5.1 Primary Analysis

Exact confidence intervals will be constructed on the probability that a subject has a primary safety endpoint. Safety will be examined separately in the vaccine and placebo groups.

9.5.2 Secondary Analyses

For the secondary analyses, descriptive statistical methods (mean, median, 25th-75th percentile, range, confidence intervals) and graphical summaries will be used.  Summaries will be made separately for the two groups of subjects (vaccine recipients and placebo recipients) for each time point of measurement. In addition, the cumulative number of subjects in each group who developed an immunologic response will be summarized, by time point.

Analyses of the immunologic measures in part 1will be restricted to time points while subjects were receiving antiretroviral treatment. Analyses of the viral load setpoint will be restricted to subjects who withdraw HAART and have a viral load setpoint as defined in 9.2.2. Treatment arms will be compared by the Wilcoxon rank-sum test for the viral load setpoint, the total ELISPOT response and the total ICS response. A supplemental analysis of the viral load setpoint will additionally include any subjects who withdrew HAART but then re-initiated HAART prior to week 12 based on the protocol-defined viral load criteria; for these subjects, the average of the last two log10 viral loads off-HAART will be used for analysis. Due to the number of comparisons, an inflated Type I error is acknowledged for these exploratory immunologic analyses.

Associations between the immunologic measures and the virologic response after HAART withdrawal will also be examined. In addition to the viral setpoint, two other aspects of the virologic response of interest are the peak viral load after HAART withdrawal and the time to confirmed viral load ≥ 50 copies/mL. In addition, the time to re-initation of HAART after withdrawal of treatment will be summarized.

# DATA COLLECTION AND MONITORING AND ADVERSE EXPERIENCE REPORTING

## 10.1 Records to Be Kept

Case report forms (CRF) will be provided for each subject. Subjects must not be identified by name on any CRFs. Subjects will be identified by the patient identification number (PID) and study identification number (SID) provided by the AACTG Data Management Center upon randomization.

## 10.2 Role of Data Management

10.2.1 Instructions concerning the recording of study data on CRFs will be provided by the AACTG Data Management Center. Each AACTU is responsible for keying the data in a timely fashion.

10.2.2 It is the responsibility of the AACTG Data Management Center to assure the quality of computerized data for each AACTG study. This role extends from protocol development to generation of the final study databases.

## 10.3 Clinical Site Monitoring and Record Availability

10.3.1 Site monitors under contract to the National Institute of Allergy and Infectious Diseases (NIAID) will visit participating clinical research sites to review the individual subject records, including consent forms, CRFs, supporting data, laboratory specimen records, and medical records (physicians’ progress notes, nurses’ notes, individuals’ hospital charts), to ensure protection of study subjects, compliance with the protocol, and accuracy and completeness of records. The monitors also will inspect sites’ regulatory files to ensure that regulatory requirements are being followed and sites’ pharmacies to review product storage and management.

10.3.2 The investigator will make study documents (e.g., consent forms, drug distribution forms, CRFs) and pertinent hospital or clinic records readily available for inspection by the local IRB, the site monitors, the Food and Drug Administration (FDA), the NIAID, the Office for Human Research Protections (OHRP).

## 10.4 Serious Adverse Experience (SAE) Reporting

This protocol follows intensive reporting requirements.

Serious adverse experiences must be documented on the Division of AIDS Serious Adverse Experience (SAE) Reporting Form and submitted to the DAIDS Regulatory Compliance Center SAE Office and are defined in the current DAIDS *Serious Adverse Experience (SAE) Reporting Manual.*

# HUMAN SUBJECTS

## 11.1 Institutional Review Board (IRB) Review and Informed Consent

This protocol and the informed consent document (Appendix III) and any subsequent modifications will be reviewed and approved by the IRB or ethics committee responsible for oversight of the study. A signed consent form will be obtained from the subject (or parent, legal guardian, or person with power of attorney for subjects who cannot consent for themselves, such as those below the legal age). The consent form will describe the purpose of the study, the procedures to be followed, and the risks and benefits of participation. A copy of the consent form will be given to the subject, parent, or legal guardian, and this fact will be documented in the subject’s record.

## 11.2 Subject Confidentiality

All laboratory specimens, evaluation forms, reports, and other records that leave the site will be identified by coded number only to maintain subject confidentiality. All records will be kept locked. All computer entry and networking programs will be done with coded numbers only. Clinical information will not be released without written permission of the subject, except as necessary for monitoring by IRB, the FDA, the NIAID, the VRC, the OHRP, or the supporter’s designee.

## 11.3 Study Discontinuation

The study may be discontinued at any time by the IRB, the NIAID, the VRC, the FDA, or other government agencies as part of their duties to ensure that research subjects are protected.

# PUBLICATION OF RESEARCH FINDINGS

Publication of the results of this trial will be governed by ACTG policies.

# BIOHAZARD CONTAINMENT

As the transmission of HIV and other blood-borne pathogens can occur through contact with contaminated needles, blood, and blood products, appropriate blood and secretion precautions will be employed by all personnel in the drawing of blood and shipping and handling of all specimens for this study, as currently recommended by the Centers for Disease Control and Prevention and the National Institutes of Health.

All infectious specimens will be transported using packaging mandated in the Code of Federal Regulations, 42 CFR Part 72. Please also refer to individual carrier guidelines, e.g., Federal Express, Airborne Express, for specific instructions.

# 14.0 REFERENCES

1. Palella FJ, Delaney KM, Moorman AC, et al. Declining morbidity and morality among patients with advanced human immunodeficiency infection. New Engl J Med 1998; 338: 853-860.
2. Borrow P, Lewicki H, Hahn BH, et al. Virus-specific CD8+ cytolytic T-lymphocyte activity associated with control of viremia in primary human immunodeficiency virus type 1 infection. J Virol 1994; 68: 6103-10.
3. Koup RA, Safrit JT, Cao Y, et al. Temporal association of cellular immune responses with the initial control of viremia in primary human immunodeficiency virus type 1 syndrome. J Virol 1994; 68: 4650-55.
4. Pantaleo G, Demarest JF, Soudeyns H, et al. Major expansion of CD8+ T cells with a predominant Vβ usage during the primary immune response to HIV. Nature 1994; 370: 463-7.
5. Musey L, Hughes J, Schacker T, et al. Cytotoxic-T-cell responses, viral load, and disease progression in early human immunodeficiency virus type 1 infection. New Engl J Med 1997; 337: 1267-74.
6. Altman JD, Moss PAH, Goulder PJR, et al. Phenotypic analysis of antigen-specific T lymphocytes. Science 1996; 274: 94-96.
7. Ogg GS, Jin X, Bonhoeffer S, et al. Quantitation of HIV-1-specific cytotoxic T lymphocytes and plasma load of viral RNA. Science 1998; 279: 2103-2106.
8. Schmitz JE, Kuroda MJ, Santra S, et al. Control of viremia in simian immunodeficiency virus infection by CD8+ lymphocytes. Science 1999; 283: 857-60.
9. Oxenius A, Price DA, Easterbrook PJ, et al. Early highly active antiretroviral therapy for acute HIV-1 infection preserves immune function of CD8+ and CD4+ T lymphocytes. Proc Natl Acad Sci USA 1997; 3382-3387.
10. Rosenberg ES, Billingsley JM, Caliendo AM, et al. Vigorous HIV-1-specific CD4+ T cell responses associated with control of viremia. Science 1997; 278: 1447-50.
11. Rosenberg ES, Altfeld M, Poon SH, et al. Immune control of HIV-1 after treatment of acute infection. Nature 2000; 407: 523-6.

14.0 REFERENCES (cont’d)

1. Lori F, Lewis MG, Xu J, et al. Control of SIV rebound through structured treatment interruptions during early infection. Science 2000; 290: 1591-93.
2. Altfeld M, Rosenberg ES, Shankarappa R, et al. Cellular immune responses and viral diversity in individuals treated during acute and early HIV-1 infection. J Exp Med 2001; 193: 169-180.
3. Tang DC, Devit M, Johnson SA. Genetic immunization is a simple method for eliciting an immune response. Nature 1992; 356: 152-154.
4. Ulmer J., Donnell, JJ, Parker SE, et al. Heterologous protection against influenza by injection of DNA encoding a viral protein. Science 1993; 259: 1745-1749.
5. Wolff JA, Malone RW, Williams P, et al. Direct gene transfer into mouse muscle *in vivo*. Science 1990; 247: 1465-1468.
6. Barouch DH, Santra S, Schmitz JE, et al. Control of viremia and prevention of clinical AIDS in rhesus monkeys by cytokine-augmented DNA vaccination. Science 2000; 290: 486-492.
7. Egan MA, Charini WA, Kuroda MJ, et al. Simian immunodeficiency virus (SIV) *gag* DNA-vaccinated rhesus monkeys develop secondary cytotoxic T-lymphocyte responses and control viral replication after pathogenic SIV infection. J Virol 2000; 74: 7485-95.
8. Shiver JW, et al. Replication-incompetent adenoviral vaccine vector elicits effective anti-immunodeficiency-virus immunity. Nature 2002; 415: 331-5.
9. Barouch DH, Kunstman J, Kuroda MJ, et al. Eventual AIDS vaccine failure in a rhesus monkey by viral escape from CTL. Nature 2002; 415: 335-9.
10. Amara RR, Villinger F, Altman JD, et al. Control of a mucosal challenge and prevention of clinical AIDS in rhesus monkeys by a multiprotein DNA/MVA vaccine. Science 2001; 292: 69-74.
11. MacGregor RR, Boyer JD, Ugen KE, et al. First human trial of a DNA-based vaccine for treatment of human immunodeficiency virus type 1 infection: safety and host response. J Infect Dis 1998;178: 92-100.

14.0 REFERENCES (cont’d)

1. Wang R, Doolan DL, Le TP, et al. Induction of antigen-specific cytotoxic T lymphocytes in humans by a malaria DNA vaccine. Science 1998; 282: 476-480.
2. Le TP, et al. Safety, tolerability and humoral immune responses after intramuscular administration of a malaria DNA vaccine to healthy adult volunteers*.* Vaccine 2000; 18: 1893-1901.
3. Tacket CO, et al. Phase 1 safety and immune response studies of a DNA vaccine encoding hepatitis B surface antigen delivered by a gene delivery device*.* Vaccine 1999; 17: 2826-2629.
4. Moore JP, Parren PW, Burton DR. Genetic subtypes, humoral immunity, and human immunodeficiency virus type 1 vaccine development. J Virol 2001;75: 5721-29.
5. Emini EA. A potential HIV-1 vaccine using a replication-defective adenoviral vaccine vector. In 9th Conference of Retroviruses and Opportunistic Infections. Seattle, Washington, 2002.
6. Korber B, et al., HIV Molecular Immunology 2000. 2000, Los Alamos, NM: Theoretical Biology and Biophysics Group.
7. Hel Z, Venzon D, Poudyal M, et al. Viremia control following antiretroviral treatment and therapeutic immunization during primary SIV251 infection of macaques. Nat Med2000; 6:1140-1146.
8. Ortiz GM., Wellons M., Brancato J., et al. Structured antiretroviral treatment interruptions in chronically HIV-1-infected subjects. Proc Nat Acad Sci USA. 2001; 98(23):13288-13293.
9. Coombs, RW, Speck CE, Hughes JP, et al. Association between culturable human immunodeficiency virus type 1 (HIV-1) in semen and HIV-1 RNA levels in semen and blood: evidence for compartmentalization of HIV-1 between semen and blood. J Infect Dis 1998; 177 (2): 320-30.
10. Garcia F., Plana M., Ortiz GM., et al. The virological and immunological consequences of structured treatment interruptions in chronic HIV-1 infection. AIDS. 2001;15 (9):F29-F40.

###### APPENDIX I

###### Definition of Antiretroviral Syndrome

Case definition must include all of the following:

1. Occurs within 8 weeks after cessation of ART.
2. Documented fever (temperature >38.5°C)
3. At least two major criteria
4. At least two minor criteria
5. No other cause for signs and symptoms identified

Major criteria (must be present for >10 days):

A. Pharyngitis

B. Fatigue

C. Morbilliform rash

D. Myalgia/arthralgia

E. Lymphadenopathy

F. Subjective fever as reported by patient (may be intermittent)

Minor criteria (must be present for >10 days):

G. Headache

H. Nausea/vomiting

I. Oral ulceration

J. Meningismus/aseptic meningitis

K. Night sweats

L. Thrombocytopenia (platelets <150,000)

M. Leukopenia (WBC <4,000)

N. Genital ulcers

O. Malaise

P. Abdominal pain

Q. Weight loss (loss of >5% of body weight)

###### APPENDIX II

SPECIMEN COLLECTION, PROCESSING, STORAGE, AND SHIPMENT

1.0 OVERVIEW

All specimens will be collected per Section 6.0, Schedule of Evaluations. Collection requirements are below. Processing details are located in sections 3.0 and 4.0 listed below. All specimens generated from this protocol must be labeled, stored and shipped according to the Laboratory and Data Management System (LDMS) guidelines unless otherwise specified.

2.0 TABLE:

| Assay/Procedure | Tube Type or Specimen Type and Quantity | Derivative | Processing Instructions | Aliquots/Storage | Shipping Address and Frequency |
| --- | --- | --- | --- | --- | --- |
| VIROLOGY |  |  |  |  |  |
| HIV-1 RNA PCR  UltraSensitive Roche Monitor test | 1 (5 mL) EDTA  BLD | Plasma | None. | 2 x 1mL PL2 | Ship real time to Johns Hopkins Virology Laboratory. |
| HIV-1 genotypic analysis | 1 (5 mL) EDTA  BLD | Plasma | None. |  | Ship real time at room temperature to Harvard ACTU Virology Laboratory (D. Kuritzkes) |
| IMMUNOLOGY |  |  |  |  |  |
| Cellular & humoral responses (ELISPOT, ICS, antibody assays) | 10 (10 mL) EDTA lavender-top tubes at weeks 6, 10, 24.  15 (10 mL) EDTA lavender-top tubes at pre-entry, entry, & weeks 26, 34, 42, 50, 60, 72, & prem. disc. | PBMC, Plasma | Ficoll blood according to ACTG specimen processing guide. Freeze PBMC viably according to Immunology Consensus Protocol in approx 10-15 (10x106 cell) aliquots. Freeze 10-15 (1 mL) aliquots of plasma. | 10-15 (10x106 cell) aliquots of frozen PBMC, and 10-15 (1 mL) aliquots of frozen plasma | Ship frozen to AACTG Specimen Repository |
| Real time immunology assays (CD4/CD8-Depleted ELISPOT, Tetramer, LPA, phenotyping of central & effector memory CTL by flow cytometry) | 8 (10 mL) EDTA lavender-top tubes at week 6, 24  10 (10 mL) EDTA lavender-top tubes at pre-entry, entry, and weeks 10, 26, 34, 42, 50, 60, 72, and premature disc. | PBMC | None |  | Ship real time at room temperature by same day or overnight carrier to Harvard ACTU Immunology Laboratories (4-5 tubes to D. Barouch, 4-5 tubes to E. Rosenberg) |
| CD4/CD8 | 1 (5 mL) EDTA lavender-top tube | PBMC | None |  | Send to a lab that possesses both CLIA certification (or equivalent) and DAIDS IQA flow lab certification. |

3.0 VIROLOGY CONSENSUS METHODOLOGY LINKS

- Virology Manual for HIV Laboratories: <http://www.niaid.nih.gov/daids/vir_manual/>
- ACTG Processing Guide Index: http://aactg:cure@aactg.s-3.com/pub/download/SpecimenProcessingGuide.pdf

4.0 IMMUNOLOGY CONSENSUS METHODOLOGY LINKS

- 3-Color Flow Cytometry Consensus Panel: http://aactg:cure@aactg.s-3.com/pub/download/imm/advflow_grid.PDF
- PBMC Cryopreservation Consensus Method: http://aactg:cure@aactg.s-3.com/pub/download/vir/freezingprotocol.pdf

IMMUNOLOGY NON-CONSENSUS ASSAY METHODS

- See Protocol-Specific Lab Sheet (PSLS)

###### APPENDIX III

DIVISION OF AIDS

ADULT AIDS CLINICAL TRIALS GROUP (AACTG)

SAMPLE INFORMED CONSENT

A5187 FINAL Version 1.0, 9/5/03

A PHASE I/II CLINICAL TRIAL TO EVALUATE THE SAFETY AND IMMUNOGENICITY OF THE HIV-1 DNA VACCINE VRC-HIVDNA009-00-VP (GAG-POL-NEF-MULTICLADE ENV) IN HIV-1-INFECTED SUBJECTS TREATED DURING ACUTE INFECTION

SHORT TITLE FOR THE STUDY: A5187, Version 1.0, 9/5/03, A Phase I**/**II Trial of VRC-HIVDNA009-00-VP.

INTRODUCTION:

You are being asked to take part in this research study because you are infected with HIV. This study is sponsored by the National Institutes of Health (NIH). The doctor in charge of this study at this site is: (insert name of Principal Investigator). Before you decide if you want to be a part of this study, we want you to know about the study.

This is a consent form. It gives you information about this study. The study staff will talk with you about this information. You are free to ask questions about this study at any time. If you agree to take part in this study, you will be asked to sign this consent form. You will get a copy to keep.

WHY IS THIS STUDY BEING DONE?

The mainpurpose of this study is to test an investigational vaccine that was developed against HIV infection to see if it is safe in HIV-infected people. Vaccines are substances used to try to create immune responses to infections such as HIV.Another goalof this study is to see if this vaccine creates an immune response to HIV. A third goal is to see what happens to the HIV viral load (the amount of HIV in blood) when anti-HIV drugs are stopped, and if there is any effect of the vaccine on HIV viral load. Participants will receive either the investigational vaccine or a placebo, which is a salt solution that does not contain any vaccine, medicine, or drugs. The responses of the participants receiving the vaccine will be compared to those receiving the placebo. This study will not be providing anti-HIV drugs.

In this study, the vaccine will be given to people by injections (shots) in the upper arms. The investigational HIV vaccine in this study is called VRC-HIVDNA009-00-VP and is not yet approved by the Food and Drug Administration (FDA). This vaccine is made from the DNA of four HIV proteins, not from the HIV virus itself. DNA serves as nature’s instructions for protein production in the body. Proteins are natural substances that the body uses as building blocks. In this study, the DNA will be injected into a muscle and will instruct the body to make a small amount of some of the proteins that are found in HIV.

This vaccine has been given to a small number of humans who are not infected with HIV and the vaccine appears safe so far although the final results are not available at this time. You will be told of any new information learned during this study that might cause you to change your mind about staying in the study. At the end of this study, you will be told when study results may be available and how to learn about them.

WHAT DO I HAVE TO DO IF I AM IN THIS STUDY?

Screening

You will need to come to the clinic within 30 days before entry into the study to see if you qualify to be in the study. At the screening visit, you will read and sign this consent form. Also, about 2 tablespoons of blood will be drawn for HIV viral load (the amount of HIV in your blood), CD4+ cell count (the number of white blood cells that fight infection), other routine blood tests, and screening for hepatitis B, hepatitis C, syphilis, and HLA typing. HLA typing is a genetic test of markers of the immune system. This test is done in this study to help follow specific types of immune responses.In addition you will give a urine sample for a routine urine test. You will be given the results of the CD4+ cell count, viral load, and routine safety tests as they become available. You will have a physical exam performed to see if you qualify to enter this study. You will be asked questions about your medical history and what medicines you are taking and have taken in the past. This visit may take up to approximately 2 hours to complete.

If you are a woman, you will have a pregnancy test done (blood or urine); about 1 teaspoon of blood or a urine sample will be given in the clinic for the pregnancy test. You will receive the results of any pregnancy test as soon as they are available.

Pre-entry

You will need to come to the clinic again within 14 days before entry into the study for additional tests. You will have about 11 tablespoons of blood drawn for routine safety tests, viral load, CD4+ cell count, and immune response tests.This visit may take up to 1 hour to complete.

If you are a woman, you will have a pregnancy test done (blood or urine); about 1 teaspoon of blood or a urine sample will be given in the clinic for the pregnancy test. You will receive the results of any pregnancy test as soon as they are available.

Entry

If you decide to be part of the study and meet the study requirements, you will be randomized (assigned by chance, like flipping a coin) to one of two groups. This means you will have an equal chance of being assigned to one of two groups. Neither you nor the research staff will know which group that you are in. One group will receive the vaccine, the other group will receive a placebo. The injections will be given into the upper arm (deltoid) muscle using a needleless system called the Biojector 2000. This hand-held system delivers the vaccine through the skin without the use of a needle. This system has FDA clearance for delivering vaccine injections into muscles. You will continue to take your anti-HIV drugsduring the entire time you are in the first part of the study.

At entry you will:

- Have a physical exam
- Have about 12 tablespoons of blood drawn to test your viral load and immune responses and other routine safety tests. You will be given the results of the CD4+ cell count, viral load and routine safety tests as they become available.
- Give a urine sample.
- Have a blood or urine pregnancy test done, if you are a woman who can become pregnant. You will receive the result of the pregnancy test.
- Receive your first vaccine or placebo injection.

During the study, Part 1 (weeks entry through 30)

You will receive a vaccine or placebo at the entry visit and weeks 4, 8, and 24. These visits may take up to 4 hours each. After receiving each injection, you will be observed for 60 minutes in the clinic. You will also take and record your temperature the same evening and for 2 days following the injection, and record any symptoms you experience. You will be evaluated by telephone 1-2 days after vaccination by staff in the clinic, and if you have any reaction other than mild (minimal pain or tenderness at the injection site), you will be asked return to the clinic within 48 hours unless the symptoms are resolving or have resolved.

At weeks 2, 4, 6, 8, 10, 16, 24, 26, and 30 you will come to the clinic to:

- Have a brief physical exam
- Have about 1-8 tablespoons of blood drawn to test your viral load, CD4+ cell count, immune responses, and other routine safety tests. You will be given the results of the CD4 cell count, viral load and routine safety tests as they become available.
- Give a urine sample.
- Have a blood or urine pregnancy test done, if you are a woman who can become pregnant, at visit weeks 4, 8, and 24. This will also be done at any time you may suspect you are pregnant. You will receive the result of the pregnancy test.

During the study, Part 2 (weeks 30 through 72)

At week 30 you will stop taking all of your anti-HIV drugs,unless you have stopped receiving the vaccine for safety reasons or pregnancy. If you are taking any non-nucleoside reversetransciptase inhibitors (NNRTI) (for example, Rescriptor, Sustiva, and Viramune), you will be switched to protease inhibitors 1 week before discontinuation of therapy or you or your doctor have the option to discontinue your NNRTI drugs 24-72 hours before discontinuing the remainder of your anti-HIV drugs. The reason for this is that these drugs tend to stay in the body for a longer period of time. You will come to the clinic every 2 weeks from weeks 30-52 and then monthly through the end of the study. Each visit should take approximately two hours. At each of these visits you will have a brief physical exam. In addition you will give a urine sample for a routine urine test. At most visits, you will give about 1 to 12 tablespoons of blood for some or most of the tests done at entry. These include routine safety tests, pregnancy test, CD4+ cell count, viral load, and immunologic testing. You will be given the results of the CD4+ cell count, viral load, pregnancy test, and routine safety tests as they become available. Your viral load and CD4+ cell counts will be carefully monitored.You will be asked to restart your anti-HIV drugs if your viral load stays very high for more than eight weeks or if your CD4+ cell count goes too low. If you and your doctor decide you should restart anti-HIV drugs, you and your doctor can choose any combination of anti-HIV drugs that you want. Before you restart your anti-HIV drugs, you will have a viral load and CD4+ cell count. In addition, testing for resistance of HIV to anti-HIV drugs will be done that may predict how you respond to anti-HIV treatment. The results of this test will not be made available to you. You should call the clinic for an appointment BEFORE you start any anti-HIV drugs.

Stored Specimens

Some of your blood will be stored (with usual protectors of identity) and used for immunologic and virologic testing that is required for this study.

Some of your blood that is left over after all required study testing is done may be stored (with usual protectors of identity) and used for AACTG approved HIV-related research.

Early Discontinuation/Withdrawal

If you stop taking the vaccine before week 24, you will be asked to come to the clinic for a physical exam, give a urine sample, and have about 12 tablespoons of blood drawn to test your viral load and immune responses and other routine safetytests. You will be given the results of the CD4+ cell count, viral load, and routine safety tests as they become available. You will then continue to be followed for safety purposes. You will not be required to stop your anti-HIV drugs at week 30, however you may do so after discussing this with your doctorunless you have stopped taking the vaccine because of safety reasons or pregnancy.

HOW MANY PEOPLE WILL TAKE PART IN THIS STUDY?

About 20 people will take part in this study.

HOW LONG WILL I BE IN THIS STUDY?

You will be in this study for about 72 weeks (about 1½ years).

WHY WOULD THE DOCTOR TAKE ME OFF THIS STUDY EARLY?

The study doctor may need to take you off the study early without your permission if:

- the study is cancelled by the U.S. Food and Drug Administration (FDA), National Institutes of Health (NIH), Vaccine Research Center (VRC),or the site’s Institutional Review Board (IRB). (An IRB is a committee that watches over the safety and rights of research subjects.)
- a Study Monitoring Committee (SMC) recommends that the study be stopped early (A SMC is an outside group of experts who monitor the study.)
- you are not able to attend the study visits or follow the instructions as required by the study

The study doctor may also need to take you off the study vaccine without your permission if:

- continuing the study vaccine may be harmful to you
- you need a treatment that you may not take while on the study
- you are not able to take the study vaccine as required by the study
- you become pregnant or begin breastfeeding.

If you must stop taking the study vaccine before the study is over, the study doctor may ask you to continue to be part of the study and return for some study visits and procedures.

WHAT ARE THE RISKS OF THE STUDY?

Injection Risks

It is possible that you may have some side effects from the injections. We will be using a needle-less system (Biojector 2000) to deliver the injections.A needle-less system uses the pressure of carbon dioxide, instead of a needle to inject the vaccine into the muscle. You may experience mild discomfort from the injections. There may be stinging, arm discomfort, pain, soreness, redness, and swelling. There is a risk of fainting and a very small chance of infection at the injection site. You will be asked to record and report any side effects you experience. You may need to make extra visits to the clinic for evaluation of side effects. You may use over-the-counter (nonprescription) pain medications, if needed.

DNA Vaccine Risks

The possible risks for vaccines in general include fever, chills, rash, aches and pains, nausea, headache, dizziness, and fatigue. We know these side effects can occur with other vaccines. They can occur whether you receive the test vaccine or the placebo in this study. If you do have a reaction, this does not mean you received the test vaccine. The side effects don't usually last long. As with all vaccines or drugs, you could have an immediate allergic reaction, including a rash, hives, or even difficulty breathing. Allergic reactions can be life threatening; therefore, the clinic staff will watch you for one hour after each immunization. There may be other side effects, even serious ones that we don't know about yet. Therefore, it is important that you report any side effects to the clinic staff as soon as they occur.

Since the particular vaccine in this study has only been given to a small number of humans so far, all the possible risks or side effects are not known. Animals have tolerated doses similar to those to be given in this study without problems. Other HIV-1 DNA vaccines have been given to humansin other studies starting as early as 1995. These vaccines appear to be well tolerated, although we do not have long-term follow-up information for more than three years for people participating in these studies.

Possible risks related to DNA vaccines include muscle damage, or antibodies (proteins made by the body’s immune system in response to a foreign substance) to DNA leading to illness. Other potential risks include insertion of the vaccine DNA into the body's DNA (leading to cancer) or into the DNA of a bacteria or virus in your body. None of these possible risks of DNA vaccines have been seen in laboratory tests or in animals or humans so far, but you need to be aware of these possible risks. It is important to know that after nearly a thousand humans have received DNA vaccines, the tolerability and safety record has been excellent so far. During the study, regular blood tests and check-ups will be performed to monitor these possible side effects.

Additional Vaccine Risk

If you receive the vaccine, you may not be able to participate in another investigational HIV vaccine study in the future.

Risks from Blood Drawing

Blood drawing may cause pain and bruising and, rarely, infection at the place where the blood is taken. Sometimes drawing blood causes people to feel lightheaded or even faint.

Risks from HLA Typing

There are few risks related to HLA typing. The greatest risk is to your privacy. It is possible that if others found out information about you that is learned from tests (such as information about your genes), it could cause you problems with family members (having a family member learn about a disease that may be passed on in families or learning who is the true parent of a child) or problems with getting a job or insurance.

#### Risks of Treatment Interruption

The long-term effects of temporarily stopping anti-HIV medications are not yet known. The following are some of the possible risks associated with treatment interruption.

Stopping anti-HIV drugs may lead to virus rebound and drug resistance. “Virus rebound” means that the HIV viral load comes back or “rebounds” to detectable levels. “Drug resistance” means that an anti-HIV drug no longer works against HIV. Drug resistance is the greatest risk of stopping and restarting medications several times. This may be of extra concern if you are currently taking any drugs called nonnucleoside reverse transcriptase inhibitors, or NNRTIs (for example, Rescriptor, Sustiva, and Viramune). Because NNRTI drugs tend to linger in the body longer than other drugs, this could increase the risk of making the virus resistant to that drug or other drugs.

It is possible that your CD4 cell levels may go down and that your HIV disease may progress more quickly after anti-HIV medications are stopped temporarily. It is also possible that the number of cells in which the virus remains latent (hidden or resting) may increase.

ARE THERE RISKS RELATED TO PREGNANCY?

It is not known if the vaccine in this study harms unborn babies. If you are having sex that could lead to pregnancy, you must agree not to become pregnant or make a woman pregnant.

Some of the drugs that you take to treat HIV may make some birth control drugs less effective. This type of birth control is given by pills, shots, or placed on orunder the skin. You must use a different method or an additional method of birth control.

You and your partner must use reliable birth control that you discuss with the study staff. You must continue to use birth control until 6 months after stopping study vaccine.

If you can become pregnant, you must have a pregnancy test before you enter this study. The test must show that you are not pregnant. If you think you may be pregnant at any time during the study, tell your study staff right away. The study staff will talk to you about your choices.

WHAT IF I BECOME PREGNANT DURING THIS STUDY?

If you become pregnant during the study, you will be asked if you wish to be followed in the study, but you will not receive additional HIV vaccine injections or begin the anti-HIV drug treatment interruption in the study. There are no anticipated additional risks to you. You and your physician will decide what anti-HIV drug combination would be best for you to continue during your pregnancy.

If you choose to stay in the study, you will continue to have safety evaluations, including routine safety tests, CD4 counts, viral loads, and physical exams, on the same schedule as described earlier in this consent. You will have about 2-3 teaspoons of blood drawn at each study visit for these tests. You may not continue to participate in this study if you choose to breastfeed your baby.

This study will not provide care related to your pregnancy, the delivery of your baby, or the care of your baby. You must arrange for your care and your baby's care outside of this study. This study will not provide your baby any anti-HIV drugs.

The possible risks to the unborn baby from the vaccine are stated above in the risks of the DNA vaccine. Long-term follow-up is recommended for a baby whose mother takes anti-HIV drugs during pregnancy. The study staff will talk to you about long-term follow-up and the possibility of enrolling your baby in a long-term follow-up study.

If your baby is injured as a result of you participating in this study, your baby will be given immediate treatment for his/her injuries. The cost of this treatment will be charged to you or your insurance company.

ARE THERE BENEFITS TO TAKING PART IN THIS STUDY?

If you take part in this study, there may be a direct benefit to you, but no guarantee can be made. It is also possible that you may receive no benefit from being in this study. Information learned from this study may help others who have HIV.

WHAT OTHER CHOICES DO I HAVE BESIDES THIS STUDY?

Instead of being in this study you have the choice of:

- treatment with prescription drugs available to you
- treatment with experimental drugs, if you qualify
- no treatment

Please talk to your doctor about these and other choices available to you. Your doctor will explain the risks and benefits of these choices.

WHAT ABOUT CONFIDENTIALITY?

We will do everything we can to protect your privacy. In addition to the efforts of the study staff to help keep your personal information private, we have gotten a Certificate of Confidentiality from the U.S. Federal Government. This certificate means that researchers cannot be forced to tell people who are not connected with this study, such as the court system, about your participation. Also, any publication of this study will not use your name or identify you personally.

People who may review your records include: the U.S. Food and Drug Administration (FDA), (insert Name of Site) IRB, National Institutes of Health (NIH), study staff, study monitors, Vaccine Research Center (VRC) and their designees. Having a Certificate of Confidentiality does not prevent you from releasing information about yourself and your participation in the study.

Even with the Certificate of Confidentiality, if the study staff learns of possible child abuse and/or neglect or a risk of harm to yourself or others, we will be required to tell the proper authorities.

WHAT ARE THE COSTS TO ME?

Taking part in this study may lead to added costs to you and your insurance company. In some cases it is possible that your insurance company will not pay for these costs because you are taking part in a research study.

During this study you will continue your anti-HIV therapy, however the drugs will not be provided by the study. You must pay for them through some other manner, such as your insurance.

WHAT HAPPENS IF I AM INJURED?

If you are injured as a result of being in this study, you will be given immediate treatment for your injuries. The cost for this treatment will be charged to you or your insurance company. There is no program for compensation either through this institution or the National Institutes of Health (NIH). You will not be giving up any of your legal rights by signing this consent form.

WHAT ARE MY RIGHTS AS A RESEARCH SUBJECT?

Taking part in this study is completely voluntary. You may choose not to take part in this study or leave this study at any time. You will be treated the same no matter what you decide.

We will tell you about new information from this or other studies that may affect your health, welfare, or willingness to stay in this study. If you want the results of the study, let the study staff know.

WHAT DO I DO IF I HAVE QUESTIONS OR PROBLEMS?

For questions about this study or a research-related injury, contact:

- name of the investigator or other study staff
- telephone number of above

For questions about your rights as a research subject, contact:

- name or title of person on the Institutional Review Board (IRB) or other organization appropriate for the site
- telephone number of above

SIGNATURE PAGE

If you have read this consent form (or had it explained to you), all your questions have been answered and you agree to take part in this study, please sign your name below.

Participant’s Name (print) Participant’s Signature and Date

Participant’s Legal Guardian (print) Legal Guardian’s Signature and Date

(As appropriate)

Study Staff Conducting Study Staff Signature and Date

Consent Discussion (print)

Witness’ Name (print) Witness’s Signature and Date

(As appropriate)
